# Modification of boron nitride nanocages by titanium doping results unexpectedly in exohedral complexes

**DOI:** 10.1038/s41467-019-12877-0

**Published:** 2019-10-28

**Authors:** Ruyi Li, Yang Wang

**Affiliations:** School of Chemistry and Chemical Engineering, Yangzhou University, 225002 Yangzhou, Jiangsu China

**Keywords:** Chemical bonding, Structure prediction, Carbon nanotubes and fullerenes, Macromolecules and clusters

## Abstract

Despite their early experimental production and observation, the unambiguous molecular structures of metal-containing boron nitride (BN) nanocages still remain mysterious. It has been commonly assumed that this family of compounds has the metal atom confined inside the cage, just like their isoelectronic cousins, carbon metallofullerenes do. Here, we demonstrate that Ti(BN)_*n*_ ($n$ = 12–24) complexes have, unexpectedly, an exohedral structure instead of an endohedral one, which could be verified by collision-induced dissociation experiments. The predicted global minimum structures exhibit some common bonding features accounting for their high stability, and could be readily synthesized under typical conditions for generating BN nanoclusters. The Ti doping dramatically changes not only the cage topology, but the arrangement of B and N atoms, endowing the resultant compounds with potential for ${\mathrm{CO}}_{2}$ capture and nitrogen fixation. These findings may expand or alter the understanding of BN nanostructures functionalized with other transition metals.

## Abstract (web summary)

Although isolated experimentally, the molecular structures of metal-containing boron nitride cages are still unknown. Here the authors show via DFT calculations that externally bound complexes of boron nitride fullerenes doped with a single titanium atom are strikingly more stable than the endohedral ones.

## Introduction

As the closest isoelectronic analog of carbon, boron nitride (BN) can likewise be shaped into a great diversity of nanoforms in various dimensions, such as nanocages, nanotubes, nanosheets, and nanoporous frameworks^1–4^. These thermally and chemically highly stable compounds have a wide range of potential applications in material^3–5^ and biomedical^6,7^ sciences. BN fullerenes, in particular, have been synthesized by electron irradiation^8,9^ or arc-melting^10,11^ methods, with their chemical compositions and cage-like structures identified by time-of-flight mass spectrometry and transmission electron microscopy. They are envisioned as promising materials for hydrogen storage^12,13^, metal-free catalysis^14^, molecular sensors^15^, and natural gas separation^16^.

In a stable BN cage, B and N atoms are alternately connected so that only N–B bonds are present and relatively much weaker N–N or B–B bonds^17^ are avoided. The consequent equal stoichiometric numbers for B and N were confirmed by electron energy loss spectroscopy^9^. For the same reason, unlike carbon fullerenes whose cage framework is usually built of pentagons and hexagons, a BN fullerene consists of squares, hexagons^18^, and sometimes octagons for larger cages^19^. The presence of any odd-membered rings, like pentagons or heptagons, would inevitably introduce N–N and B–B bonds. Among a large number of possible isomers, the energetically most favorable BN cages tend to have all squares staying away from each other so as to minimize the strain energy induced by contact of these small rings. This leads to the so-called isolated square rule^18^, an analog of the well-known isolated pentagon rule for carbon fullerenes^20^. Recently, much attention has been paid to the intriguing fact that this latter rule is often violated when a carbon cage encapsulates a metal atom or a metal-containing cluster, due to strong metal-cage interactions^21–23^. More generally, the observed cage topology (viz., the connectivity between atoms) of many endohedral carbon metallofullerenes differs dramatically from that of the corresponding neutral empty fullerenes^21–23^. A natural question arises: whether the same behavior occurs when a BN fullerene combines with a metal atom.

Experimentally, BN cages containing a single metal atom have been produced by arc-melting synthesis, where the formation of Fe(BN)_36_^24^, La(BN)_36_^25^ and Y(BN)_*n*_ ($n$ = 36, 37, 48)^11^ species was confirmed by mass spectrometry and high-resolution electron microscopy. However, due to lack of crystal structure determination, the unambiguous, atomically resolved structures of metal-doped BN fullerenes still remain unclear. The same problem was encountered for relatively small carbon metallofullerenes ${\mathrm{MC}}_{2n}$ (M = Ti, Zr, U; $n$ = 14–25), which were detected in gas-phase mass spectra^26^. Nevertheless, with the aid of quantum chemistry calculations, the molecular structures of Ti@${\mathrm{C}}_{2n}$ have been convincingly identified^27^. Among them, the above-mentioned modification of cage form upon combining with the metal was also manifested^27^. One important but perhaps overlooked fact is that all Ti-doped carbon fullerenes are endohedral complexes with the metal atom confined inside the cage. This conclusion has been extended, by implication, to BN metallofullerenes, and the observed molecules in experiments were interpreted as endohedral species^11,24,25^. Although unproven, it has even become a commonly accepted premise upon which many studies on metal-doped BN cages are based^28–31^. As a matter of fact, the electron microscopy images for metal-containing BN cages^11,24,25^ were two dimensional projections of nanostructures^30^, and therefore could not really tell whether the metal stayed inside or outside of the cage.

In this work, we show that BN fullerenes doped with a single Ti atom in general have an *exohedral* structure, at least for cage sizes comparable to their experimentally observed carbon counterparts, Ti@${\mathrm{C}}_{2n}$^26^. DFT calculations clearly demonstrate that the externally bound complexes of Ti(BN)_*n*_ are strikingly more stable than the endohedral ones, by an energy of the order of 100 kcal mol^−1^. As strongly contrasted with carbon fullerenes, it is highly exergonic for a BN cage to attach a Ti atom from outside, whereas encaging it is totally hindered from a thermodynamic point of view. This suggests that exohedral Ti(BN)_*n*_ complexes can most likely be produced by high-temperature synthesis^32,33^ like arc-melting, where global minimum structures on the free energy surface are usually formed. Furthermore, changes in cage structure take place upon doping with a single Ti atom, even more drastic than the case of carbon fullerenes. The doped cage contains a few pentagons and even heptagons, the unwelcome rings that are always absent in a pristine BN fullerene. As we will see, such a variation in cage framework, along with notable rearrangement of B and N atoms, allows for a maximum metal-cage interaction, featured by the formation of four strong Ti–N bonds. Based on the understanding of the extraordinary stability of these uncovered complexes, some simple topological rules are established, enabling us to find, among a vast number of possibilities, the most stable structures of Ti-doped BN fullerenes that are likely to be observed in future experiments.

## Results

### Global minimum structures of Ti(BN)_19_

**Fig. 1:**
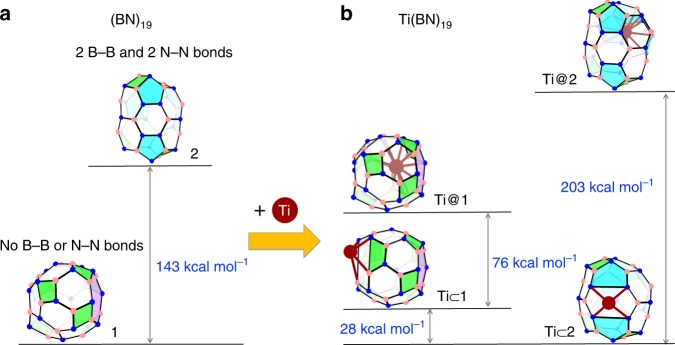
Relative energies of two isomers of pristine cages (BN)_19_ and of their Ti-doped complexes. **a** Isomer **1** is the lowest energy form of (BN)_19_, consisting of 7 squares, 13 hexagons, and 1 octagon, with no B–B or N–N bond present. Isomer **2** contains 3 squares, 6 pentagons, and 12 hexagons. The presence of two B–B and two N–N bonds makes the latter isomer lie significantly higher in energy than the former. **b** Conversely, when doped with a single Ti atom, cage **2** becomes much more stable than cage **1**, and gives the global minimum structure of Ti(BN)_19_. For both cages, the exohedral complex, Ti $\subset$ (BN)_19_, has substantially lower energy than the corresponding endohedral one, Ti@(BN)_19_. All relative energies (including zero-point energy correction) have been obtained from DFT calculations. Boron, nitrogen, and titanium atoms are indicated by pink, blue, and dark red circles, respectively. Squares, pentagons, hexagons, and octagons are shown in green, cyan, white, and magenta, respectively

We have considered the possibility of doping (BN)_*n*_ cages with a Ti atom, given the fact that so far only a single Ti, Zr, or U atom has been experimentally inserted into small carbon fullerenes^26^. The choice of Ti would be of particular interest in practice, since its nontoxic nature permits safe experiments for macroscopic synthesis^26^. To start off, we selected a medium-sized Ti(BN)_19_, considering the size range of experimentally observed Ti@${\mathrm{C}}_{2n}$ (*n* = 14–25), for a thorough investigation. According to the present and previous^19^ DFT calculations (see Methods), the lowest energy isomeric form of prinstine (BN)_19_ is cage **1** (see Fig. 1a), which consists of 7 squares, 13 hexagons, and 1 octagon, with alternately linked B and N atoms. In comparison, cage isomer **2** shown in Fig. 1a contains 3 squares, 6 pentagons, and 12 hexagons and has thus, inevitably, two B–B and two N–N bonds. As expected, the latter cage is significantly less stable (by 143 kcal mol^−1^) than the former. However, when they are doped with a Ti atom the situation is reversed: the lowest energy isomer of Ti(BN)_19_ is the one with the Ti atom bound outside cage **2** (hereafter symbolized by Ti $\subset$ **2**), while isomer Ti $\subset$ **1** lies 28 kcal mol^−1^ higher than Ti $\subset$ **2**, as shown in Fig. 1b. Yet, a more unexpected observation is that for both cages, binding a Ti atom outside the cage is substantially more favorable in energy than encapsulating the metal inside; as shown in Fig. 1b, the relative energy of the endohedral Ti@(BN)_19_ with respect to the corresponding exohedral Ti $\subset$ (BN)_19_ is as high as 76 and 203 kcal mol^−1^, respectively, for cage **1** and **2**. This is a remarkably surprising result since it is well-known that carbon metallofullerenes are generally endohedral complexes with enclosed metal atoms or clusters (at least for cage sizes as small as ${\mathrm{C}}_{28}$),^21,26^ and for this reason it has been commonly assumed that their BN analogs have likewise an endohedral structure^11,24,25,28–31^.

**Fig. 2:**
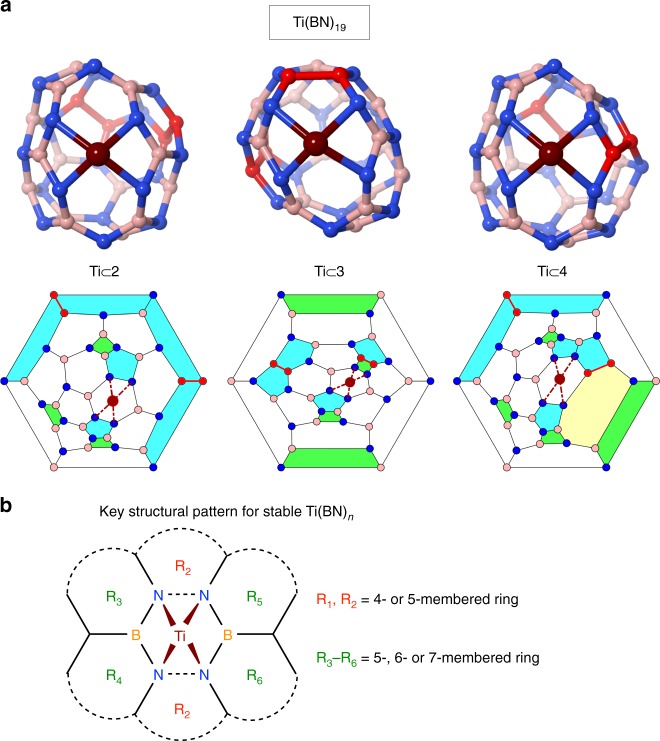
Global minimum energy structures of Ti(BN)_19_, revealing common bonding features. **a** Molecular structures (upper panel) and Schlegel diagrams (lower panel) of the three global minimum isomers (almost degenerate within an energy difference of 0.7 kcal mol^−1^). Boron, nitrogen, and titanium atoms are depicted in pink, blue, and dark red, respectively. The two B–B bonds in each molecule are highlighted in red. In Schlegel diagrams, Ti–N bonds are indicated by dashed lines, and squares, pentagons, hexagons, and heptagon are shown in green, cyan, white, and yellow, respectively. **b** The key structural pattern present in all stable Ti-doped (BN)_*n*_ cages. The Ti atom is located above a hexagonal ring, where four N atoms sit on two parallel sides and two B atoms at opposite corners. To achieve higher stability, the top and bottom rings (${\mathrm{R}}_{1}$ and ${\mathrm{R}}_{2}$) adjacent to the doped hexagon are preferably squares or pentagons, while the four lateral neighboring rings (${\mathrm{R}}_{3}$–${\mathrm{R}}_{6}$) opt for pentagons, hexagons, or heptagons

By a systematic and exhaustive search of low-energy isomers of Ti(BN)_19_ (see Methods and Supplementary Methods 1–3), we have found, aside from the aforementioned Ti $\subset$ **2**, other two global minimum structures, Ti $\subset$ **3** and Ti $\subset$ **4** (see Fig. 2a). The energy difference between the three is less than 0.7 kcal mol^−1^. Although differing in cage topology (cage **4** even has a heptagon, see the Schlegel diagrams in Fig. 2a), the three isomers share some common bonding features responsible for their high stability. As shown in Fig. 2a, the Ti atom is exohedrally bonded to the four N atoms located on a hexagonal ring, which, before combined with Ti, has two N–N bonds on the parallel sides and four B–N bonds on the other (for simplicity, hereafter we refer to such a hexagon as the *key hexagon*). Upon doping, the two parallel N–N bonds break up so as to form four tetragonal pyramid-shaped Ti–N bonds (with N–Ti–N angles of about 75°and 90°). Note that the cleavage of N–N bonds enables the N atoms to stably coordinate to the Ti while maintaining the delocalized $\pi$ bonding between N and B atoms. Because of the two N–N linkages on the key hexagon, the global minimum structures have inevitably two B–B bonds in the rest part of the cage (see the bonds in red color in Fig. 2a), whose strength is much weaker and therefore destabilizes the system to some extent. Nonetheless, this destabilization is surpassed by the formation of four strong Ti–N bonds. The Ti–N bond lengths in Ti $\subset$ **2** are 1.95–1.98 Å, even slightly shorted than those in the ${\mathrm{TiN}}_{n}^{+}$ clusters recently produced by laser ablation and observed by mass spectrometry.^34^ Besides, additional stability is gained in the complex owing to the reduced strain energy, as the modified cage by Ti doping (form **2**) contains more pentagons and less squares than the pristine one (form **1**, see Fig. 1).

### Structural and bonding features of stable Ti(BN)_19_ isomers

A closer inspection of the neighboring rings surrounding the key hexagon reveals more rules for determining low energy Ti(BN)_*n*_ isomers. By examining nearly 2500 possible candidate structures of Ti $\subset$ (BN)_19_ containing a key hexagon, we have found that all low-lying isomers comply with the following rules. First, the neighboring rings that share an N–N side with the key hexagon (i.e., the top and bottom rings shown in Fig. 2b, R_1_ and R_2_) are either a square or a pentagon. The strain induced by these smaller rings can facilitate the opening of the N–N bonds and hence the formation of Ti–N bonds. Moreover, the other four neighboring rings of the key hexagon (i.e., the lateral rings shown in Fig. 2b, R_3_–R_6_) prefer to be a pentagon, or a hexagon, or a heptagon. The larger, less strained lateral rings seem to help sustain the N–B bonds in the key hexagon upon hosting a Ti atom. As shown in Table 1, these structural characteristics are clearly evidenced in the low-lying isomers of Ti $\subset$ (BN)_19_ (within 10 kcal mol^−1^ with respect to the global minimum). Many more examples are provided in Supplementary Note 3 and Supplementary Table 6.

**Table 1:** Structural patterns in the low-lying isomers of Ti $\subset$ (BN)_19_

Isomer^a^	RE^b^	R_1_^c^	R_2_^c^	R_3_^c^	R_4_^c^	R_5_^c^	R_6_^c^
Ti $\subset$ **2**	0.0	5	5	6	6	6	6
Ti $\subset$ **3**	0.3	4	5	6	6	6	6
Ti $\subset$ **4**	1.1	5	5	6	6	5	7
Ti $\subset$ **5**	3.4	5	5	6	6	6	6
Ti $\subset$ **6**	5.9	4	4	6	6	6	6
Ti $\subset$ **7**	6.1	5	5	6	5	6	6
Ti $\subset$ **8**	6.7	5	5	6	6	6	6
Ti $\subset$ **9**	8.0	5	5	6	5	6	6
Ti $\subset$ **10**	8.8	5	5	6	6	6	5
Ti $\subset$ **11**	9.1	5	5	6	6	6	6
Ti $\subset$ **12**	9.3	5	5	6	6	6	6
Ti $\subset$ **13**	9.6	4	5	6	5	6	6
Ti $\subset$ **14**	9.7	5	5	6	6	6	6
Ti $\subset$ **15**	9.8	5	5	6	6	7	5

^a^Isomers are numbered in ascending order of their relative energies

^b^Relative energies in kcal mol^−1^, without zero-point energy correction

^c^Neighboring rings surrounding the key hexagon as described in Fig. 2b. The values 4, 5, 6, and 7 represent, respectively, square, pentagon, hexagon, and heptagon

**Fig. 3:**
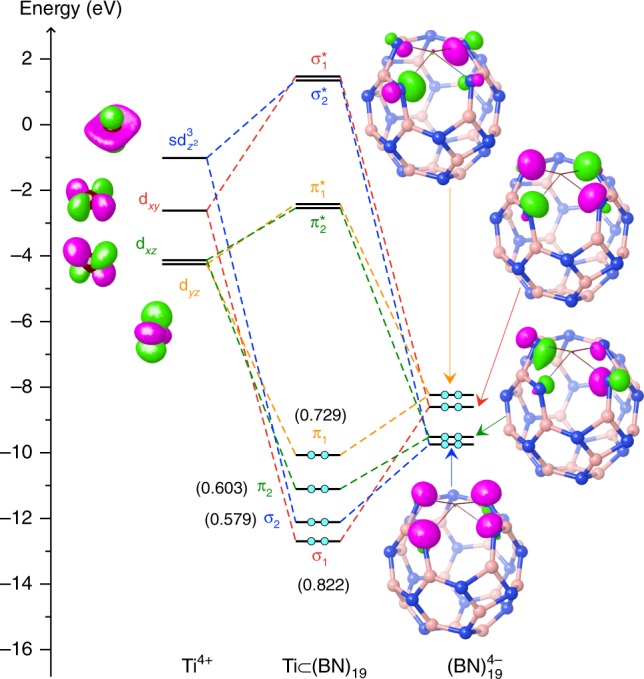
Orbital correlation diagram unraveling the nature of the metal-cage bonding in Ti $\subset$ **2**. The analysis is based on the maximum bonding fragment orbital approach^35,36^. Each of the Ti’s atomic orbitals (left panel) is paired up with one of the cage’s fragment orbitals (right panel), leading to a pair of bonding and anti-bonding orbitals of the complex (middle panel). The energy levels of the associated fragment orbitals and complex orbitals are connected by dashed lines in the same color. Electrons are shown by cyan filled circles. Orbital surfaces are depicted using an isovalue of 0.10. The Wiberg bond orders^37^ in parentheses indicate that all these four bonding interactions are essentially covalent. According to the aufbau principle^35^, this orbital diagram suggests an oxidation state of +4 for Ti

To gain further understanding of why the key hexagon is essential for the stability of Ti(BN)_*n*_ complexes, we have analyzed orbital interactions between the metal and the cage, based on the maximum bonding fragment orbital approach^35,36^. Figure 3 presents the orbital correlation diagram for the lowest energy complex, Ti $\subset$ **2**, revealing four principal metal-cage bonding interactions (each with a Wiberg bond order^37^ greater than 0.5). We can see that the three leading interactions (${\sigma }_{1}$, ${\pi }_{1}$, and ${\pi }_{2}$) are between Ti’s *d* orbitals (${d}_{xy}$, ${d}_{yz}$, and ${d}_{xz}$, respectively) and the cage’s symmetry-adapted group orbitals, the latter coming mostly from the four N atoms of the key hexagon. The last major bonding, ${\sigma }_{2}$, is resulted from the *sd*^3^ hybridized orbital of Ti interacting with the group orbital from the N atoms. The significant values of bond orders (about 0.6–0.8) for these interactions indicate four typical polarized covalent bonds^35^ formed between the metal and the cage, reflecting the maximum bonding ability acquired by the tetravalent Ti, which would account for the high stability of the complex Ti $\subset$ **2**. Further, in order to achieve a maximum overlap between the fragment orbitals, especially as required by the symmetry of the strongest bonding orbital, ${\sigma }_{1}$, the four Ti–N bonds adopt preferably a tetragonal pyramidal conformation, as mentioned above and illustrated in Fig. 2. It is worth noting that, in accord with the aufbau principle^35^, the effective oxidation number of Ti in this compound is +4, as expected for this group 4 transition metal.

**Fig. 4:**
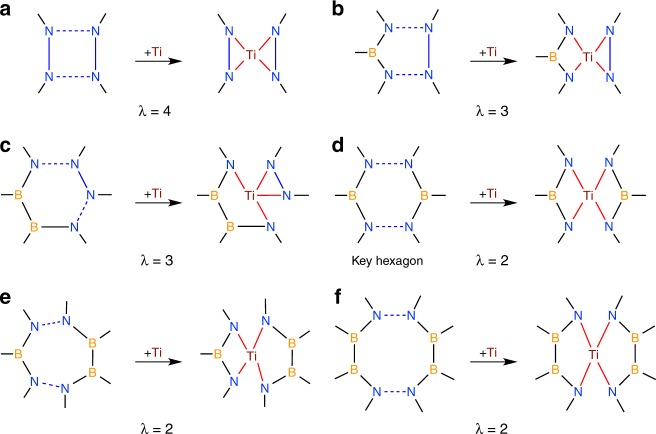
N–N bond cleavage and Ti–N bond formation schemes upon Ti doping of a BN cage. The additions of a Ti atom to the following faces of the BN cage are considered: **a** A square with  $\lambda$= 4, where $\lambda$ denotes the number of N–N/B–B bonds. **b** A pentagon with $\lambda$ =3. **c** A hexagon with four N atoms in tandem ( $\lambda$= 3). **d** A key hexagon with two parallel N–N bonds ($\lambda$ = 2). **e** A heptagon with two separated N–N bonds ($\lambda$ = 2). **f** An octagon with two parallel N–N bonds ($\lambda$ = 2). The N–N bonds to be broken are indicated by blue dashed lines, while the subsequently formed Ti–N bonds are shown by red solid lines

Isomers with less than four Ti–N bonds are much higher in energy, such as Ti $\subset$ **1** shown in Fig. 1b (see Supplementary Note 4 and Supplementary Fig. 5 for more examples), since a Ti–N bond is almost twice as strong as a Ti–B bond (their bond energy being 114 and 65 kcal mol^−1^ (ref. ^17^), respectively). Furthermore, binding the Ti atom with four N atoms from a square or a pentagon will introduce more energetically unfavorable N–N and B–B bonds (see Fig. 4a, b, where the number of these bonds, $\lambda$, is indicated), which would significantly destabilize the system. Likewise, we can rule out the possibility of adding the Ti to a hexagon site where the four N atoms are consecutively placed, as indicated in Fig. 4c. As for additions to a heptagonal or octagonal ring, although it is possible to host a tetracoordinated Ti while maintaining only two N–N/B–B connections (see Fig. 4e, f), calculations show that Ti $\subset$ (BN)_19_ with such a heptagon (octagon) ligand lie at least 22.5 (85.1) kcal mol^−1^ in energy above the global minimum (see Supplementary Figs. 6 and 7 for more examples). The main reason is that a larger ring like heptagon or octagon is more flexible and thus more easily subject to deformation induced by the strain in the cage. Consequently, the four Ti–N bonds deviate noticeably from the ideal tetragonal pyramidal configuration, with distorted bond angles and the four N atoms out of coplanarity (see Supplementary Note 5). Based on the above discussion, it is reasonable to speculate that the key hexagon provides a chemically favorable site to accommodate a Ti atom, constituting the essential feature of stable Ti-doped BN cages.

**Fig. 5:**
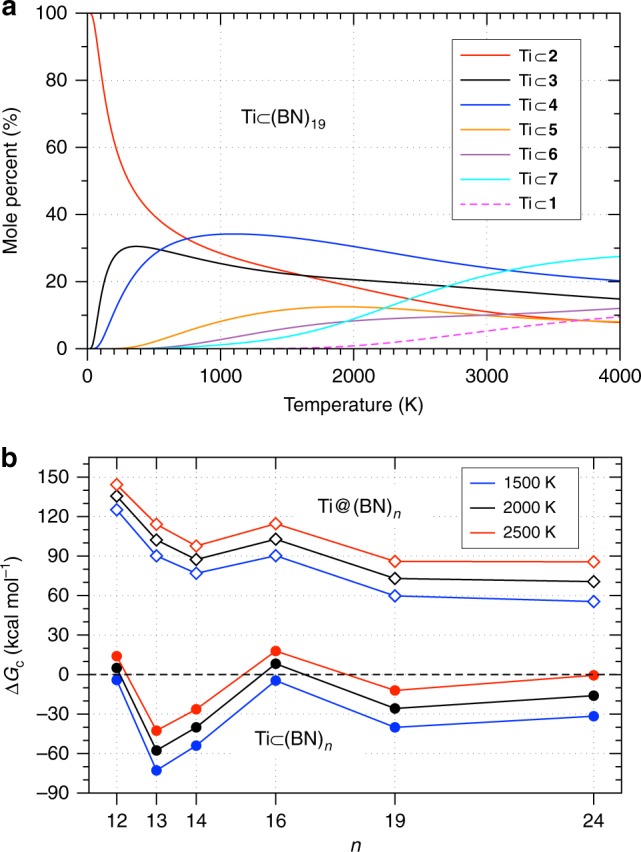
Relative concentrations and complexation free energies of Ti(BN)_*n*_ at various temperatures. **a** DFT predicted mole fractions of the major products by a thermodynamically controlled synthesis of Ti(BN)_19_, as a function of temperature up to 4000 K. The solid lines correspond to the six lowest energy isomers and the dashed line denotes the isomer with cage form **1**. **b** Gibbs free energies of complexation of Ti(BN)_*n*_ ($n=12,13,14,16,19,24$), $\Delta {G}_{{\rm{c}}}$, defined by Eq. (1) in the text, at 1500, 2000, and 2500 K (indicated in blue, black, and red, respectively), the typical temperatures for producing BN nanocages^32,33^. Solid circles and empty diamonds represent, respectively, the exohedral and endohedral complexes. Note that the exohedral products presented here are the lowest Gibbs free energy isomer at a given temperature, while the endohedral products have the cage form that corresponds to the lowest energy isomer of pristine cages (see Supplementary Note 7, Supplementary Table 9 and  Supplementary Fig. 14 for specific structures). All results in **a** and **b** are obtained at a pressure of 1 atm

At this point, we can propose simple structural rules for stable Ti-doped BN cages, as follows: (1) the cage contains one key hexagon with appropriate neighboring rings to host the Ti atom, as described in Fig. 2b; (2) it has only two B–B bonds; and (3) it obeys the isolated square rule^18^. Violations to the latter two rules lead to higher energy isomers, as exemplified in Supplementary Tables 7 and 8. By applying these rules to all possible cage isomers of (BN)_19_ (including regioisomers with different arrangement of B and N atoms), we ended up with 1065 candidates. Subsequent prescreening and refinement DFT calculations allowed us to predict the lowest energy isomers of Ti(BN)_19_ that could be produced in experiments. For thermodynamically controlled gas-phase generation such as under arc-melting conditions^11,24,25^, we have predicted the temperature-dependent mole fractions of major products of Ti(BN)_19_ on the basis of Maxwell-Boltzmann distribution^38^ (see Supplementary Note 8). For clarity, Fig. 5a only shows the relative concentrations of six major products (from Ti $\subset$ **2** to Ti $\subset$ **7**, labeled in ascending order of energy), along with that of isomer Ti $\subset$ **1** for comparison. Their relative energies are presented in Table 2 and molecular structures are shown in Supplementary Fig. 12. At typical temperature range (1500–2500 K) for producing BN nanocages^32,33^, isomer Ti $\subset$ **4** with a heptagonal ring is the most abundant product, followed by Ti $\subset$ **3**. Despite being the lowest energy isomer at 0 K, Ti $\subset$ **2** is only the third major product at synthesis temperatures up to ~2400 K. Beyond that temperature, other relatively higher energy isomers (especially Ti $\subset$ **7**) become more competitive. In comparison, the product formed with the lowest energy pristine cage, Ti $\subset$ **1** (indicated by the dashed line in Fig. 5a), would be obtained in a very low yield during the whole temperature range of interest.

**Table 2:** Relative energies (in kcal mol^−1^) of the ten lowest-energy isomers of Ti(BN)_*n*_, in comparison with isomers Ti $\subset$ **1**, Ti@**1,** and Ti@**2**

Isomer	Ti(BN)_12_	Ti(BN)_13_	Ti(BN)_14_	Ti(BN)_16_	Ti(BN)_19_	Ti(BN)_24_
Ti $\subset$ **1**	4.3	38.7	9.0	9.9	27.7	20.5
Ti@**1**	123.6	169.9	138.7	92.3	103.4	88.2
Ti $\subset$ **2**	0.0	0.0	0.0	0.0	0.0	0.0
Ti@**2**	187.7	297.1	267.1	212.1	202.7	182.6
Ti $\subset$ **3**	2.1	3.5	6.7	1.0	0.3	0.7
Ti $\subset$ **4**	2.1	7.1	12.7	3.4	0.7	1.3
Ti $\subset$ **5**	5.9	17.6	12.9	10.4	3.2	2.0
Ti $\subset$ **6**	7.3	18.8	13.3	10.5	5.7	2.7
Ti $\subset$ **7**	8.0	19.1	14.1	11.0	5.9	4.6
Ti $\subset$ **8**	8.2	19.7	15.2	11.6	6.2	5.5
Ti $\subset$ **9**	8.8	20.0	15.9	12.1	7.9	5.5
Ti $\subset$ **10**	9.1	20.3	17.1	12.2	8.3	6.0
Ti $\subset$ **11**	11.4	21.9	18.4	12.7	8.6	6.5

### Synthetic viability and possible experimental verification

Guided by the stability rules as an efficient prescreening tool, we have discovered the lowest energy structures of Ti(BN)_*n*_ of other cage sizes (see Methods and Supplementary Method 3 for detailed procedures). These structures are summarized in Supplementary Note 6 and Supplementary Figs. 8–13. The temperature-dependent mole fractions for their major products are presented in Supplementary Figs. 15–19. Similar to the case of Ti(BN)_19_, all exohedral complexes are considerably more stable than the endohedral ones, even for cages as large as (BN)_24_, as can be seen in Table 2. To explore the viability of their synthesis, we have computed, at arc-melting temperatures and a pressure of 1 atm, the Gibbs free energies of complexation, $\Delta {G}_{{\rm{c}}}$, defined as

1 $$\Delta {G}_{{\rm{c}}}=G[{{\rm{Ti(BN)}}}_{n}]-G[{\rm{Ti}}]-G[{{\rm{(BN)}}}_{n}],$$

where $G[{{\rm{Ti(BN)}}}_{n}]$, $G[{\rm{Ti}}]$, and $G[{{\rm{(BN)}}}_{n}]$ are the Gibbs free energy of the complex Ti(BN)_*n*_, of a single Ti atom and of the pristine cage (BN)_*n*_, respectively. Note that we have taken, depending on the given temperature, the lowest *free energy* isomer for (BN)_*n*_ and for Ti(BN)_*n*_, which may differ from the lowest energy form at 0 K. The solid circles in Fig. 5b present the complexation free energies of Ti(BN)_*n*_ for $n$ = 12, 13, 14, 16, 19, and 24 at temperatures 1500, 2000, and 2500 K. As we can see, all Ti $\subset$ (BN)_*n*_ complexes can be spontaneously formed at all these temperatures except for $n$ = 12 and 16, whose synthesis may not be ruled out at lower temperatures like 1500 K, or at a higher pressure^33,39^. Notably, the production of Ti $\subset$ (BN)_13_ and Ti $\subset$ (BN)_14_ are highly exergonic ($\Delta {G}_{{\rm{c}}}$ = $-57.6$ and $-40.1$ kcal mol^−1^ at 2000 K, respectively). The reason is that their pristine cage reactants are exceptionally unstable, for it is mathematically impossible to have a cage form satisfying the isolated square rule for (BN)_13_ and (BN)_14_ (ref. ^18^). This implies that these otherwise elusive BN clusters can be readily stabilized by doping with a single Ti atom.

In direct contrast, Fig. 5b also depicts the complexation free energies of endohedral products made of the lowest energy pristine cage, which are apparently all strongly endergonic at arc-melting temperatures. By comparison, their carbon counterparts exhibit the opposite behavior: for instance, at 2000 K and 1 atm, the DFT calculated $\Delta {G}_{{\rm{c}}}$ = $-3.8$ and $-37.7$ kcal mol^−1^ for Ti $\subset$ ${\mathrm{C}}_{38}$ and Ti@${\mathrm{C}}_{38}$ (ref. ^27^), respectively. This supports the fact that *endohedral* metallofullerenes are generally observed in arc-discharge production^21,26^, whereas the formation of *exohedral* species is usually precluded for carbon fullerenes unless by direct attachment of the metal to a preformed cage at relatively low temperatures^40^. The striking contrast between carbon and BN fullerenes is probably due to the following facts. In a nonpolar, homonuclear carbon fullerene, the frontier $\pi$ electrons are delocalized over the whole cage, preferring to form an also delocalized, multicenter bond with the Ti atom. And by binding the metal inside the comparatively isotropic cage, a maximum coordination number is achieved and so is maximum stability. On the other hand, a BN fullerene consists of polar bonds with much more localized valence electrons. The Ti atom seems to go for the more electronegative, N atoms other than the B atoms, leading to four Ti–N bonds, as allowed by this tetravalent metal. Since the metal-cage bonding is essentially localized, an exohedral configuration is favored, probably in order to minimize strain energy and/or to maximize orbital overlaps.

To confirm the exohedral structure of Ti(BN)_*n*_ complexes, collision-induced dissociation experiments could be carried out. Early such experiments^40^ unequivocally confirmed that the single metal atom in ${\mathrm{MC}}_{60}^{+}$ (M = Fe, Co, Ni, Cu, Rh, La) was externally bound to the carbon cage, as a result of direct detachment of the metal at relatively low excitation energies. In the present case of exohedral Ti(BN)_*n*_, collisional excitation or photoexcitation would give rise to Ti^+^ ions, leaving the BN cage neutral and intact, since the ionization potential of the latter (e.g., 8.2 eV for (BN)_13_ by DFT) is much higher than that of Ti (6.6 eV). Accordingly, one should detect only Ti^+^ ions in mass spectra, by using an excitation energy of the order of 10 eV (e.g., the computed dissociation energy of [Ti $\subset$ (BN)_13_]^+^ being 10.7 eV). Otherwise, if the metal atom is enclosed in the cage, much higher excitation energy would be required to take out the metal, and the fragmented pieces of parent cage might be observed as well.

In conclusion, we have found that doping BN fullerenes with a single Ti atom generally results in exohedral complexes instead of endohedral ones, which is unexpectedly contrary to the case of most carbon metallofullerenes. Inserting a metal atom into a fullerene molecule needs to break first the chemical bonds of the cage, requiring a sufficiently hot environment so that the metal is being enclosed during the formation of its host cage. However, DFT calculations show that at such high temperatures encapsulation of a Ti atom into a BN nanocage is thermodynamically inhibited, although it might be accomplished by resort to molecular surgery^41,42^. Conversely, exohedrally doped BN fullerenes exhibit high thermodynamic stability that makes their synthesis viable at high temperatures typical for yielding BN nanocages. We suggest that the exohedral nature of Ti(BN)_*n*_ clusters could be proved experimentally by studying the collision-induced dissociation^40^ or dissociation following photoexcitation of the complexes.

The doping with a single Ti atom can change profoundly not only the cage topology, but also the arrangement of B and N atoms. As opposed to pristine BN cages consisting of even-membered rings to avoid any B–B or N–N contacts, Ti-doped cages incorporate also pentagons and contain two B–B bonds, in order to acquire maximum bonding interaction with Ti. The energy cost paid by introducing two relatively weak B–B bonds is compensated and surpassed by the formation of four strong Ti–N bonds, as well as by the alleviation of strain in the doped cage compared to the pristine one. Interestingly, these B–B antisites have been shown to have potential for ${\mathrm{CO}}_{2}$ capture^43^ and nitrogen fixation^44^ at ambient conditions.

In view of the present results for Ti-doped BN nanocages, it is conceivable to expect similar or different implications for other BN nanostructures functionalized with other transition metals. For instance, preliminary calculations suggest that different transition metals may behave differently in modifying the structures of BN nanocages, which opens an interesting topic for future research. We hope our findings will stimulate further experimental investigations to generate, identify, and characterize this particular family of compounds.

## Methods

### DFT calculations

All DFT calculations were performed using the Gaussian 16 suite of programs^45^ at the B3LYP/6-31+G(d) level, including Grimme’s D3 dispersion correction with Becke–Johnson damping^46^. The accuracy of basis set was verified by additional calculations using the Def2-TZVP and Def2-TZVPP basis sets (see Supplementary Note 1 and Supplementary Tables 2 and 3). We also carried out some single-point calculations with the double-hybrid B2PLYP-D3/Def2-TZVP method and obtained similar relative energies and complexation energies (see Supplementary Note 2 and Supplementary Tables 4 and 5). All geometries were optimized without any constraint and confirmed to be minima on the potential energy surface via vibrational frequency analysis. For low-lying energy isomers, both singlet and triplet electronic states were taken into account, and the stability of wavefunction was verified. Relative energies were computed including zero-point energy correction.

### Procedures of search for global minimum structures

Topologies of all BN cages were constructed by the plantri program^47^, and then converted to atomic Cartesian coordinates by the embed program in the CaGe package^48^. Cage isomers with distinct atomic connectivity and regioisomers with distinct B and N distribution are uniquely identified based on the canonical labeling of atoms^49^ following the breadth-first-search numbering algorithm^50^. We systematically determined the lowest energy isomers of pristine cages, which all have alternately linked B and N atoms. To enumerate possible candidates for cages containing a key hexagon, we optimized the arrangement of B and N atoms and attained all possible isomers having the minimum number (namely two) of B–B bonds (see Supplementary Method 2 and Supplementary Figs. 1 and 2 for algorithm). The subsequent Ti $\subset$ (BN)_*n*_ complexes were calculated using the 6-31G(d) (or 3-21G for $n$ = 48) basis set (see Supplementary Figs. 3 and 4 for justification of using the 6-31G(d) basis set), allowing us to rule out a great number of high energy isomers. For endohedral complexes Ti@(BN)_*n*_, as well as Ti $\subset$ (BN)_*n*_ made of the lowest energy pristine cage, we explored all possible initial structures by placing the Ti atom at different faces of the cage and determined the corresponding lowest energy isomer. Preliminary tests showed that the Ti atom bound at vertex or bond center sites are energetically less favorable than at face centers. The detailed prescreening procedure is described in Supplementary Method 3, with the total number of considered structures summarized in Supplementary Table 1.

## Data Availability

The authors declare that the data supporting the findings of this study are available within the paper and its supplementary information files.

## Supporting Information

Supplementary Information
Peer Review File
Description of Additional Supplementary Files
Dataset 1
Dataset 2
Dataset 3
Dataset 4
Dataset 5
Dataset 6
Dataset 7
Dataset 8
Dataset 9
Dataset 10
Dataset 11
Dataset 12
Dataset 13
Dataset 14
Dataset 15
Dataset 16
Dataset 17
Dataset 18
Dataset 19
Dataset 20
Dataset 21
Dataset 22
Dataset 23
Dataset 24
Dataset 25
Dataset 26
Dataset 27
Dataset 28
Dataset 29
Dataset 30
Dataset 31
Dataset 32
Dataset 33
Dataset 34
Dataset 35
Dataset 36
Dataset 37
Dataset 38
Dataset 39
Dataset 40
Dataset 41
