# Supplementary material for: Modification of boron nitride nanocages by titanium doping results unexpectedly in exohedral complexes
Source: Nat Commun. 2019 Oct 28;10:4908. doi: 10.1038/s41467-019-12877-0 (PMC6961409; doi:10.1038/s41467-019-12877-0)
Supplement: Supplementary file 1 — Supplementary Information [file 41467_2019_12877_MOESM1_ESM.pdf]

Supplementary Information

Modification of boron nitride nanocages by titanium doping results unexpectedly in exohedral complexes

Li et al.

Contents

Supplementary Method 1: Identification of cage isomers and regioisomers of $(BN)_n$ fullerenes 3

Supplementary Method 2: Minimization of the number of B–B (N–N) bonds for a given cage topology 3

Supplementary Method 3: Procedure of search of global minimum structures of complex $Ti(BN)_n$ 5

Supplementary Note 1: Basis set convergence tests 8

Supplementary Note 2: Comparison with double-hybrid DFT calculations 10

Supplementary Note 3: Representative isomers of $Ti{\subset}(BN)_{19}$ containing a key hexagon 11

Supplementary Note 4: Isomers of $Ti{\subset}(BN)_{19}$ with less than four Ti–N bonds 15

Supplementary Note 5: Binding the Ti atom with four N atoms of a heptagon or an octagon 16

Supplementary Note 6: Lowest-energy isomers of $Ti(BN)_n$ 17

Supplementary Note 7: Lowest Gibbs free energy isomers of $Ti(BN)_n$ 24

Supplementary Note 8: Temperature-dependent mole fractions of major products of $Ti(BN)_n$ 25

Supplementary References 28

Supplementary Method 1: Identification of cage isomers and regioisomers of $(BN)_n$ fullerenes

Given a $(BN)_n$ fullerene molecule, there are two types of isomerism. First, the topology of the cage framework (i.e., the connectivity between atoms) gives a unique trivalent molecular graph, provided that all vertices (atoms) are indistinguishable. Hence, $(BN)_n$ and C_{2n} fullerenes have the same cage isomerism. To rigorously enumerate all possible cage isomers (trivalent molecular graphs), we have used the plantri program.[1] On the other hand, on top of a fixed cage topology, there are many possible ways to place n B and n N atoms, leading to the regioisomerism. For this reason, we use the letter i to denote an isomeric form of the cage connectivity and the letter j to denote a regioisomer of the BN arrangement.

Unlike classical fullerene cages, which consist of only pentagons and hexagons and can thus be readily identified using Fowler-Manolopoulos ring spiral algorithm,[2] the $(BN)_n$ cages considered in this work generally contain squares, pentagons, hexagons, and some may contain heptagons or octagons as well. Here, we have employed an algorithm[3] based on the connectivity list using the canonical labeling of vertices that follows the breadth-first-search (BFS) numbering scheme.[4,5] Using this technique, both the cage isomer and the regioisomer of BN distribution can be uniquely and readily identified.

Supplementary Method 2: Minimization of the number of B–B (N–N) bonds for a given cage topology

Supplementary Figure 1 and 2 outline the algorithm of optimization of the arrangement of B, N atoms to minimize the number of B–B and N–N bonds for a given cage topology of size $2n$. On the basis of the canonical labeling of all cage atoms,[3] we first choose a starting B atom at position iAt (running from 1 to $2n$) to generate an initial arrangement of B, N atoms. This initial guess of B, N positions, corresponding to regioisomer j_0, are deduced by placing B and N, as alternately as possible, on the cage vertices, following the connectivity list. Apparently, such a procedure does not necessarily give the optimal B, N arrangement with a minimum number of B–B (N–N) bonds. Nevertheless, it is reasonable enough for the subsequent optimization, which is to be detailed below. As shown in Supplementary Figure 1, after one step of optimization of B, N positions, usually a number of better regioisomers $\{j\}$ are generated, which have fewer B–B (N–N) bonds than the initial regioisomer j_0. Then, this better set of regioisomers $\{j\}$ will be used as new initial guess configurations and be subject to a second round of optimization, which will generate more new regioisomers possibly with fewer B–B (N–N) bonds. By repeating the same procedure for $maxIter$ iterations, we will arrive at a converged set of regioisomers with a minimum possible number of B–B (N–N) bonds. In the present work, we took a sufficiently large value of 1000 for $maxIter$ to ensure the convergence. On top of this, by running the starting B atom at all possible positions on the cage (i.e., iAt varying from 1 to $2n$), it is almost guaranteed that all regioisomers with the minimum possible number of B–B and N–N bonds are found.

Now, we describe the algorithm of minimizing the number of B–B and N–N bonds, starting from a given initial arrangement of B and N atoms. As shown in Supplementary

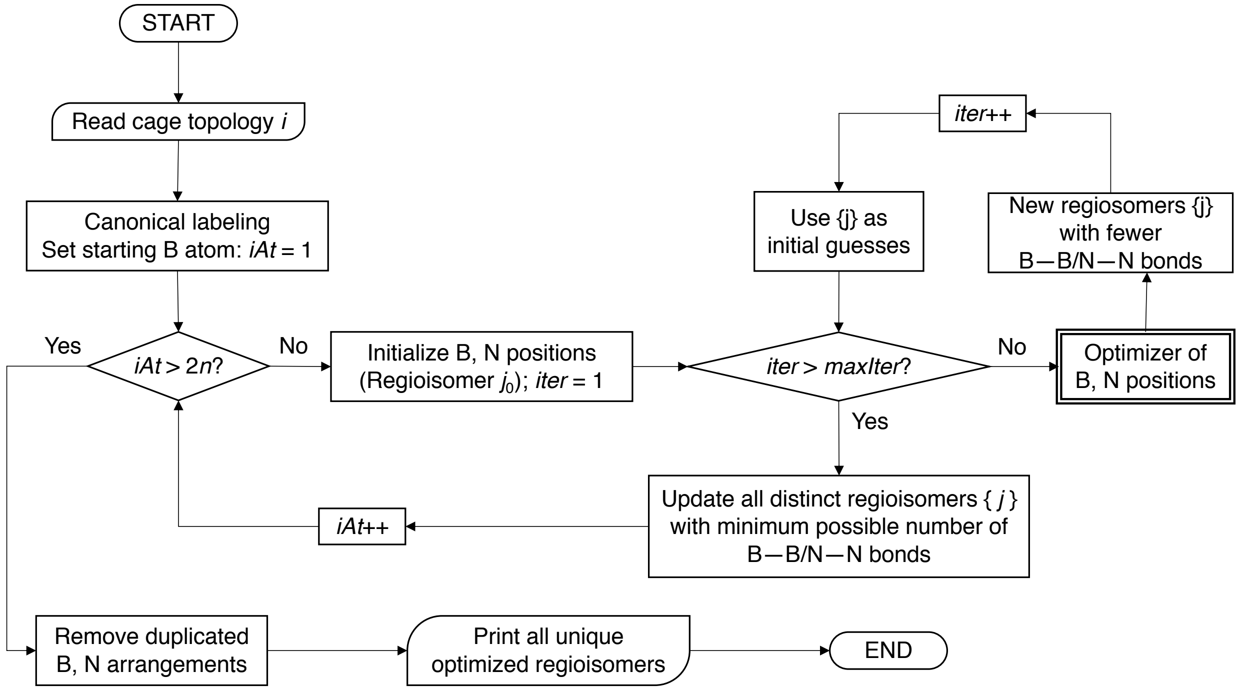

Supplementary Figure 1. Flowchart for search of possible regioisomers of $(BN)_n$ with a given cage form that contain the minimum possible number of B–B and N–N bonds. Variable iAt represents the position on the cage for the starting B atom, and $maxIter$ is the maximum number of iterations to run.

Figure 2, given a cage topology i with a regioisomer j_0, we count the initial number of B–B (N–N) bonds, denoted by N_0. By running over all possible (B, N) pairs (no matter they are bonded or not), an interchange between each pair of B, N atoms, say at positions iB and iN, respectively, is rejected if the resultant number of B–B (N–N) bonds increases; otherwise, the swap is accepted, and the corresponding B, N arrangement is kept as a new regioisomer $j(iB, iN)$. In the end, among all generated new regioisomers, topologically identical ones are removed, by using the canonical numbering technique.[3]

To check the efficacy of the algorithm, we optimized the B, N arrangement for some cages composed of squares, hexagons and/or octagons, with a random distribution of B, N atoms so that the initial number of B–B (N–N) bonds are nonzero. In all cases, the optimizations arrived at a correct arrangement with alternate B and N atoms on the cage, as expected. Similarly, we also tried some cages containing pentagons, and the optimizations succeeded to reduce the number of B–B (N–N) bonds to a reasonably small number, and sometimes being one, the minimum possible number of B–B (N–N) bonds that a pentagon-containing cage could possess. Moreover, we have tested the optimization starting from a worst scenario: the B, N atoms were initially placed in such a way that one half side of the cage was filled with all B atoms and the other half with all N atoms. The optimizations led to consistent results with those starting from a more reasonable initial guess of B, N positions.

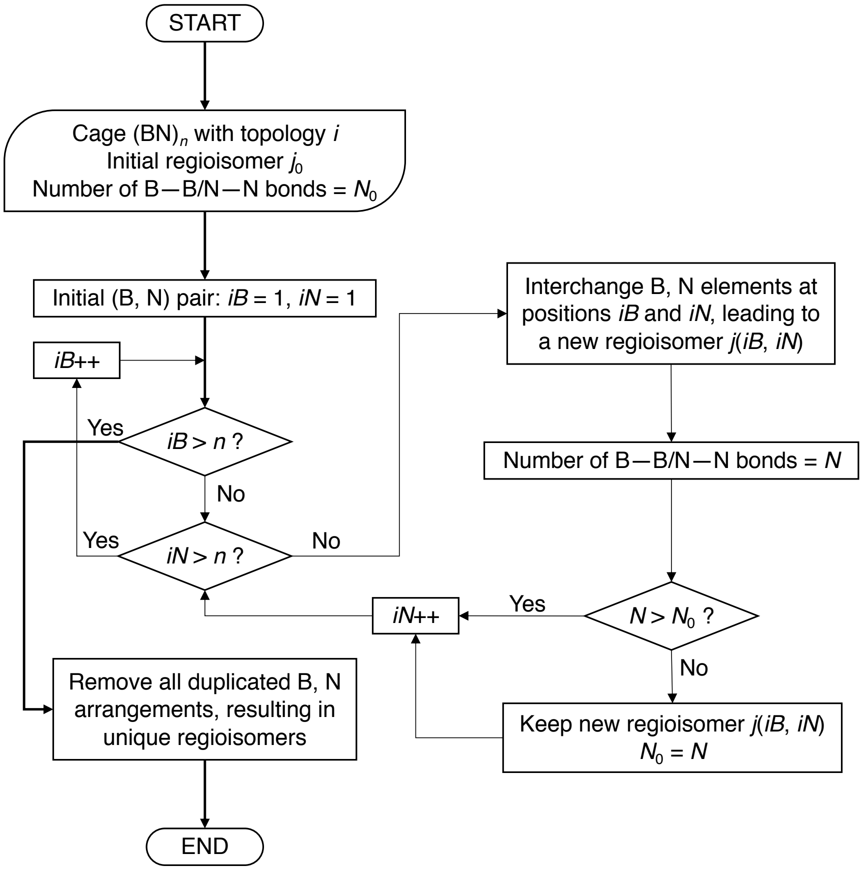

Supplementary Figure 2. Flowchart for optimization of B and N positions to minimize the number of B–B and N–N bonds, for a given cage topology i, and with a given initial B, N arrangement (regioisomer j_0) that has been generated by setting the starting B atom at position iAt after $iter$ iterations of optimization (see Supplementary Figure 1). After the optimization, a new set of better regioisomers $\{j\}$ are generated.

Supplementary Method 3: Procedure of search of global minimum structures of complex $Ti(BN)_n$

1. $Ti(BN)_{19}$

The most extensive search was conducted in the case of $Ti(BN)_{19}$. First, we considered doping the two lowest-energy cage isomers made of squares and hexagons. For each isomer, both endohedral and exohedral doping were systematically considered, by placing initially a Ti atom above all nonequivalent vertices, bond centers and face centers, followed by full geometry optimization. In the endohedral case, an additional initial guess was also taken into account by putting the Ti atom at the geometric center of the cage. In addition, we explored adding the Ti atom to the lowest-energy isomer of pristine cages of $(BN)_{19}$ (namely, **1**, consisting of an octagon, squares and hexagons), to the third lowest-energy cage isomers consisting of squares and hexagons, the five lowest-energy cage isomers consisting of squares, pentagons and hexagons, as well as the lowest energy cage isomer consisting of only pentagons

and hexagons. In these cases, only doping at faces were taken into account.

Second, we enumerated all possible $(BN)_{19}$ cage isomers that are made of squares, pentagons, hexagons, or made of squares, pentagons, hexagons and a heptagon, or made of squares, pentagons, hexagons and an octagon, and meanwhile contain a key hexagon and have two B–B bonds in the cage.

For each cage candidate, we took each of the hexagonal faces to make the key hexagon, by placing four N atoms on a pair of parallel sides and two B atoms on the rest two corners. For each hexagon, there are at most three possibilities of making the key hexagon (symmetrical equivalent regioisomers are discarded). After the initial assignation of a given key hexagon, there are $(2n-6)$ vertices left on the cage for arranging the rest $(n-2)$ B and $(n-4)$ N atoms. We then applied the optimization described in the preceding subsection to find the optimal B, N arrangements with a minimized number of B–B bonds. All generated regioisomers with more than two B–B bonds were discarded. Note that there was no constraint about the neighboring rings surrounding the key hexagon (namely, not following the rules given in Figure 2b in the text). We did not apply the isolated square rule (ISR)[6] to all these candidates except for those containing a heptagon or an octagon. Subsequently, for all generated BN cage candidates, we placed a single Ti atom above the center of the key hexagon and performed full geometry optimization for prescreening.

Lastly, we explored all possible isomers where the Ti atom is bound to a heptagon or an octagon forming four Ti–N bonds (as specified in Figure 4e,f in the text). In these cases, we only considered the ISR-obeying structures.

Note that we have also taken into account the reversed arrangement of B and N atoms if it gives a different regioisomer from the original one. Thus, we have considered about 2,500 isomers in total for $Ti(BN)_{19}$ (see Supplementary Table 1). A prescreening procedure was performed for all candidates at the B3LYP-D3/6-31G(d) level. Subsequently, all obtained isomers lying below 20 kcal mol^{-1} relative to the lowest energy were re-optimized at the B3LYP-D3/6-31+G(d) level, considering both singlet and triplet states. We also performed the B3LYP-D3/6-31+G(d) calculations for many other higher energy isomers. In total, we had 535 isomers of $Ti(BN)_{19}$ in the final refinement calculations, as shown in Supplementary Table 1.

Supplementary Table 1. The total number (N) of isomers considered in the prescreening and refinement calculations for the search of low-lying structures of $Ti(BN)_n$ (n = 12, 13, 14, 16, 19 and 24). All geometries were fully optimized at the B3LYP-D3 level of theory.

N	$Ti(BN)_{12}$	$Ti(BN)_{13}$	$Ti(BN)_{14}$	$Ti(BN)_{16}$	$Ti(BN)_{19}$	$Ti(BN)_{24}$
Prescreening	454[a]	162[a]	243[a]	804[a]	2,521[a]	6,743[b]; 79[a]
Refinement	32[c]	33[c]	38[c]	51[c]	535[c]	84[c]

[a]Using the 6-31G(d) basis set. [b]Using the 3-21G basis set. [c]Using the 6-31+G(d) basis set.

Ti(BN)$_n$ of other sizes (n = 12, 13, 14, 16, 24)

For other cage sizes considered in the present work (i.e., n = 12, 13, 14, 16, 24), we searched among all possible cage isomers built of squares, pentagons, hexagons, and a maximum number of one heptagon (or octagon) that contain a key hexagon and have two B–B bonds. Note that due to the relatively small size of the BN cages considered here ($12 \leqslant n \leqslant 24$), isomers with more than one heptagonal or octagonal ring would be rather high in energy. Also, we considered the doping at all possible faces of the lowest-energy pristine cage (i.e., denoted by **1**, following the notations throughout this work). Additionally, for n = 16 and 24, we considered binding the Ti atom on a heptagon or an octagon (as illustrated in Figure 4e,f in the text) for all possible isomers made of squares, pentagons, hexagons, and one heptagon (or one octagon) and containing two B–B bonds. All these cage candidates include both ISR and non-ISR isomers except that for n = 24 only ISR isomers were taken into account due to its much larger cage size with a huge number of possible isomers and high computational cost. We imposed no constraint on the neighboring rings surrounding the key hexagon (namely, as given by the rules described in Figure 2b in the text), except that for n = 24 the R_3–R_6 rings are assumed to be all hexagons.

All candidates were first prescreened at the B3LYP-D3/6-31G(d) level of theory, except that the 3-21G basis set was employed for n = 24 due to the high computational cost. All resulting structures lying below the lowest-energy one by 20 kcal mol^{-1} were re-optimized at the B3LYP-D3/6-31+G(d) level. To sum up, the total numbers of Ti(BN)$_n$ isomers considered in the prescreening and refinement calculations are listed in Supplementary Table 1.

Supplementary Note 1: Basis set convergence tests

To validate the 6-31+G(d) basis set employed in the present work, we did some convergence tests by comparing the results with those obtained from B3LYP-D3 calculations with larger Def2-TZVP and Def2-TZVPP basis sets. Supplementary Table 2 presents such a comparison for relative energies of pristine cages $(BN)_{19}$ and of complexes $Ti(BN)_{19}$. The numbering of cage isomers **1**–**4** is explained in the text. For their detailed molecular structures, see Figure 1 and Figure 2a in the text as well as Supplementary Figure 12. Similar comparison is made for Gibbs free energies of complexation of $Ti(BN)_{19}$ (see equation (1) in the text for definition), as shown in Supplementary Table 3. As we can see, for both relative energies and complexation free energies, the three basis sets give very similar results.

Supplementary Table 2. Relative energies (including zero-point energy correction) of pristine cages $(BN)_{19}$ and of complexes $Ti(BN)_{19}$, calculated at the B3LYP-D3 level using different basis sets. All energies are in kcal mol^{-1}.

Isomer	6-31+G(d)	Def2-TZVP	Def2-TZVPP
**1**	0.0	0.0	0.0
**2**	143.3	145.8	145.8
Ti⊂**1**	27.7	25.4	25.4
Ti@**1**	103.4	103.5	103.6
Ti⊂**2**	0.0	0.0	0.0
Ti@**2**	202.7	205.3	205.2
Ti⊂**3**	0.3	0.2	0.2
Ti⊂**4**	0.7	0.2	0.2

Supplementary Table 3. Gibbs free energies of complexation of $Ti(BN)_{19}$, calculated at the B3LYP-D3 level using different basis sets. All results are obtained at a pressure of 1 atm and temperature of 2000 K. All energies are in kcal mol^{-1}.

Isomer	6-31+G(d)	Def2-TZVP	Def2-TZVPP
Ti⊂**1**	−11.4	−11.6	−11.9
Ti@**1**	73.0	75.4	75.1
Ti⊂**2**	−23.9	−22.2	−22.5
Ti@**2**	180.1	184.5	184.1
Ti⊂**3**	−23.8	−22.1	−22.5
Ti⊂**4**	−25.8	−24.4	−24.7

To justify the 6-31G(d) basis set we used in the prescreening procedures for search of low-lying $Ti(BN)_n$ isomers, we compared the relative energies calculated at the B3LYP-D3 level with 6-31G(d) and 6-31+G(d) basis sets. As shown in Supplementary Figures 3 and 4 for systems $Ti\subset(BN)_{19}$ and $Ti\subset(BN)_{24}$, respectively, the results obtained by using the 6-31G(d) basis set are in very good agreement with those by the 6-31+G(d).

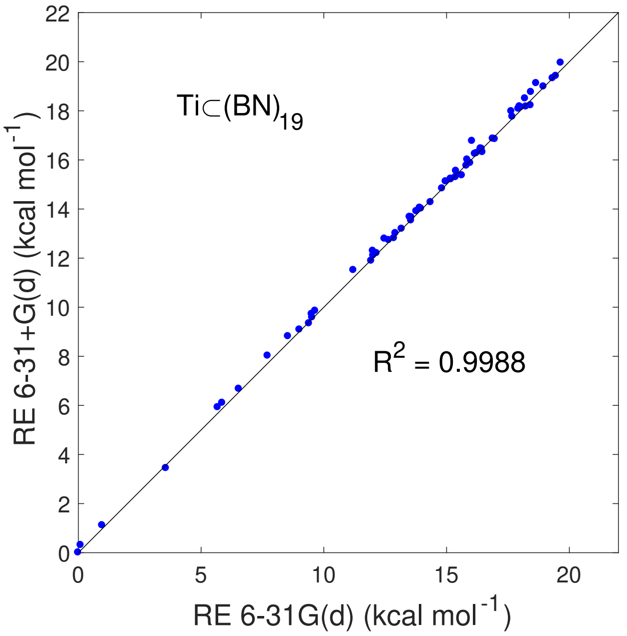

Supplementary Figure 3. Relative energies (RE, without zero-point energy correction) of the 65 lowest-energy isomers of Ti⊂$(BN)_{19}$, calculated at the B3LYP-D3 level. Results are compared between basis sets 6-31G(d) and 6-31+G(d).

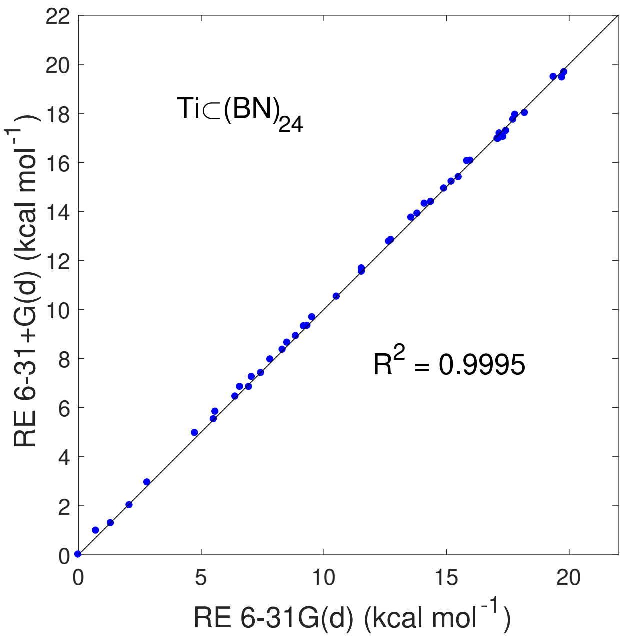

Supplementary Figure 4. Idem Fig. 3 for the 45 lowest-energy isomers of Ti⊂$(BN)_{24}$.

Supplementary Note 2: Comparison with double-hybrid DFT calculations

Here, we compare the results obtained from the B3LYP-D3/Def2-TZVPP calculations (with full geometry optimization) with those obtained using the B2PLYP-D3/Def2-TZVP [7] method. The B2PLYP-D3 is among the double-hybrid functionals with best performance for energetics of transition metal complexes.[8,9] Due to very large computational cost, for the latter method, we only performed single-point calculations based on the B3LYP-D3/Def2-TZVPP optimized geometries. The tested systems are the same as those used in the assessment in Supplementary Note 1. As we can see in Supplementary Tables 4 and 5, the B3LYP-D3/Def2-TZVPP calculated relative energies, as well as complexation energies, are in line with those computed at the B2PLYP-D3/Def2-TZVP level.[7] Note that the complexation energy of a given $Ti(BN)_{19}$ complex is defined as $\Delta E_c = E[Ti(BN)_{19}] - E[Ti] - E[(BN)_{19}]$, where $E[Ti(BN)_{19}]$, $E[Ti]$ and $E[(BN)_{19}]$ are the total energy of the complex $Ti(BN)_{19}$, of a single Ti atom and of the lowest-energy pristine cage $(BN)_{19}$, respectively.

Supplementary Table 4. Relative energies of pristine cages $(BN)_{19}$ and of complexes $Ti(BN)_{19}$, calculated at the B3LYP-D3/Def2-TZVPP//B3LYP-D3/Def2-TZVPP and the B2PLYP-D3/Def2-TZVP//B3LYP-D3/Def2-TZVPP levels. All energies are in kcal mol^{-1}.

Isomer	B3LYP-D3/Def2-TZVPP	B2PLYP-D3/Def2-TZVP
**1**	0.0	0.0
**2**	146.1	150.3
Ti⊂**1**	26.5	33.1
Ti@**1**	104.9	103.0
Ti⊂**2**	0.0	0.1
Ti@**2**	205.8	200.1
Ti⊂**3**	0.1	0.0
Ti⊂**4**	0.5	0.2

Supplementary Table 5. Complexation energies of $Ti(BN)_{19}$, calculated at the B3LYP-D3/Def2-TZVPP//B3LYP-D3/Def2-TZVPP and the B2PLYP-D3/Def2-TZVP//B3LYP-D3/Def2-TZVPP levels. All energies are in kcal mol^{-1}.

Isomer	B3LYP-D3/Def2-TZVPP	B2PLYP-D3/Def2-TZVP
Ti⊂**1**	−57.5	−48.4
Ti@**1**	20.9	21.5
Ti⊂**2**	−84.0	−81.4
Ti@**2**	121.7	118.6
Ti⊂**3**	−83.9	−81.5
Ti⊂**4**	−83.5	−81.3

Supplementary Note 3: Representative isomers of Ti⊂$(BN)_{19}$ containing a key hexagon

Low-lying isomers of Ti⊂$(BN)_{19}$

Supplementary Table 6 summarizes the relative energies and structural characteristics for low-lying isomers (within 20 kcal mol^{-1} with respect to the global minimum) of Ti⊂$(BN)_{19}$ containing a key hexagon. R_1–R_6 are the number of the corresponding neighboring rings surrounding the key hexagon in each isomer (see Figure 2b in the text for detailed description).

As we can see, all these isomers satisfy the structural rules proposed in the text, that is, the neighboring rings R_1 and R_2 are either a square or a pentagon; R_3–R_6 are either a pentagon, or a hexagon, or a heptagon. Meanwhile, it is worth mentioning that all these isomers also obey the other two stability rules: There are only two B–B bonds present in the cage; and the cage topology follows the isolated square rule.[6]

Supplementary Table 6. Relative energies (RE, in kcal mol^{-1}, without zero-point energy correction) for low-lying isomers of Ti⊂(BN)$_{19}$ (within 20 kcal mol^{-1}), calculated at the B3LYP-D3/6-31+G(d) level. R_1–R_6 are the neighboring rings surrounding the key hexagon in each isomer (see Figure 2b in the text for detailed description). The values 4, 5, 6 and 7 represent, respectively, square, pentagon, hexagon and heptagon. The cage isomers in Ti⊂2—Ti⊂66 are labeled in ascending order of the relative energy of the complexes.

Isomer	RE	R_1	R_2	R_3	R_4	R_5	R_6	Isomer	RE	R_1	R_2	R_3	R_4	R_5	R_6
Ti⊂**2**	0.0	5	5	6	6	6	6	Ti⊂**35**	15.2	5	5	6	6	6	5
Ti⊂**3**	0.3	4	5	6	6	6	6	Ti⊂**36**	15.2	5	5	6	5	6	6
Ti⊂**4**	1.1	5	5	6	6	5	7	Ti⊂**37**	15.3	4	5	6	6	6	6
Ti⊂**5**	3.4	5	5	6	6	6	6	Ti⊂**38**	15.4	5	5	6	6	6	6
Ti⊂**6**	5.9	4	4	6	6	6	6	Ti⊂**39**	15.5	4	5	6	5	6	6
Ti⊂**7**	6.1	5	5	6	5	6	6	Ti⊂**40**	15.8	5	5	6	6	6	6
Ti⊂**8**	6.7	5	5	6	6	6	6	Ti⊂**41**	15.9	5	5	6	6	6	6
Ti⊂**9**	8.0	5	5	6	5	6	6	Ti⊂**42**	15.9	4	5	6	6	6	6
Ti⊂**10**	8.8	5	5	6	6	6	5	Ti⊂**43**	16.0	5	5	7	6	6	6
Ti⊂**11**	9.1	5	5	6	6	6	6	Ti⊂**44**	16.2	5	5	6	5	6	6
Ti⊂**12**	9.3	5	5	6	6	6	6	Ti⊂**45**	16.3	5	5	6	5	6	6
Ti⊂**13**	9.6	4	5	6	5	6	6	Ti⊂**46**	16.3	5	5	6	5	6	6
Ti⊂**14**	9.7	5	5	6	6	6	6	Ti⊂**47**	16.4	5	5	5	6	6	6
Ti⊂**15**	9.8	5	5	6	6	7	5	Ti⊂**48**	16.4	5	5	6	6	6	5
Ti⊂**16**	11.5	4	5	6	6	6	6	Ti⊂**49**	16.5	5	5	6	6	5	7
Ti⊂**17**	11.9	4	5	6	6	6	6	Ti⊂**50**	16.8	5	5	4	6	6	5
Ti⊂**18**	12.1	4	5	6	6	6	6	Ti⊂**51**	16.8	5	5	6	5	6	6
Ti⊂**19**	12.2	4	5	6	6	6	6	Ti⊂**52**	16.9	5	5	6	6	6	6
Ti⊂**20**	12.3	5	5	5	6	6	6	Ti⊂**53**	17.8	4	5	6	6	6	6
Ti⊂**21**	12.7	5	5	6	5	6	6	Ti⊂**54**	18.0	5	5	6	5	6	6
Ti⊂**22**	12.8	5	5	6	6	6	5	Ti⊂**55**	18.1	5	5	6	6	6	5
Ti⊂**23**	12.8	4	5	6	6	6	6	Ti⊂**56**	18.1	5	5	6	6	5	7
Ti⊂**24**	13.0	5	5	6	6	6	5	Ti⊂**57**	18.2	5	5	6	6	6	6
Ti⊂**25**	13.2	5	5	6	6	6	6	Ti⊂**58**	18.2	5	5	6	6	5	6
Ti⊂**26**	13.5	5	5	6	5	6	6	Ti⊂**59**	18.2	4	5	6	6	6	6
Ti⊂**27**	13.7	4	5	6	6	6	6	Ti⊂**60**	18.5	4	5	6	6	6	6
Ti⊂**28**	13.7	4	5	6	5	6	6	Ti⊂**61**	18.8	5	5	6	5	5	6
Ti⊂**29**	13.9	5	5	7	5	6	6	Ti⊂**62**	19.0	4	5	6	6	6	6
Ti⊂**30**	14.0	5	5	6	6	6	6	Ti⊂**63**	19.1	5	5	6	6	5	5
Ti⊂**31**	14.0	5	5	6	6	6	6	Ti⊂**64**	19.3	5	5	6	6	6	6
Ti⊂**32**	14.3	5	5	6	6	6	6	Ti⊂**65**	19.4	4	5	6	7	6	6
Ti⊂**33**	14.8	5	5	6	6	6	6	Ti⊂**66**	20.0	5	5	6	6	5	6
Ti⊂**34**	15.1	5	5	6	6	6	6								

Key-hexagon-containing isomers that violate the isolated square rule

Supplementary Table 7 lists some Ti⊂$(BN)_{19}$ isomers that contain a key hexagon and violate the isolated square rule (ISR).[6] We can see that these ISR-violating isomers are at least 41.4 kcal mol^{-1} in energy above the global minimum. Therefore, we can rule out non-ISR structures when searching for stable Ti⊂$(BN)_{19}$ complexes.

Supplementary Table 7. B3LYP-D3/6-31G(d) calculated relative energies (RE, in kcal mol^{-1}, without zero-point energy correction) for Ti⊂$(BN)_{19}$ isomers that contain a key hexagon and violate the isolated square rule. R_1–R_6 are the neighboring rings surrounding the key hexagon in each isomer (see Figure 2b in the text for detailed description). The values 4, 5 and 6 represent, respectively, square, pentagon and hexagon. NASP is the number of adjacent square pairs in the cage framework. The numbering of isomer Ti⊂n means that it is the n-th lowest-energy isomer in the prescreening set.

Isomer	RE	R_1	R_2	R_3	R_4	R_5	R_6	NASP
Ti⊂**396**	41.4	4	5	6	6	6	6	1
Ti⊂**592**	49.7	4	5	6	6	6	6	1
Ti⊂**618**	50.5	5	5	6	5	6	5	1
Ti⊂**661**	52.3	5	5	6	5	6	5	1
Ti⊂**672**	52.9	4	5	5	6	6	6	1
Ti⊂**718**	54.9	4	5	6	6	5	6	1
Ti⊂**1468**	89.3	5	5	6	5	6	6	2
Ti⊂**1480**	90.2	5	5	6	6	6	6	2
Ti⊂**1521**	92.7	5	5	6	6	6	6	2
Ti⊂**1558**	95.0	5	5	6	6	6	6	2
Ti⊂**1883**	125.1	4	5	6	6	5	6	2

Key-hexagon-containing isomers that have more than two B–B bonds

Supplementary Table 8 shows some Ti⊂$(BN)_{19}$ isomers that contain a key hexagon and with four B–B bonds present in the cage. Note that all these isomers obey the isolated square rule. As can be seen, they are at least 113.2 kcal mol^{-1} higher in energy than the global minimum isomers, and can thus be ruled out.

Supplementary Table 8. B3LYP-D3/6-31G(d) calculated relative energies (RE, in kcal mol^{-1}, without zero-point energy correction) for Ti⊂$(BN)_{19}$ isomers that contain a key hexagon and four B–B bonds. R_1–R_6 are the neighboring rings surrounding the key hexagon in each isomer (see Figure 2b in the text for detailed description). The values 4, 5 and 6 represent, respectively, square, pentagon and hexagon. N_{B-B} is the number of B–B bonds present in the cage. The numbering of isomer Ti⊂n means that it is the n-th lowest-energy isomer in the prescreening set.

Isomer	RE	R_1	R_2	R_3	R_4	R_5	R_6	N_{B-B}
Ti⊂**1800**	113.1	5	5	6	5	5	6	4
Ti⊂**1828**	117.1	5	5	6	5	6	6	4
Ti⊂**1832**	118.0	5	5	6	5	5	6	4
Ti⊂**1853**	120.9	5	5	6	6	6	5	4
Ti⊂**1861**	121.9	5	5	6	6	6	5	4

Supplementary Note 4: Isomers of Ti⊂$(BN)_{19}$ with less than four Ti–N bonds

Supplementary Figure 5 presents examples of Ti⊂$(BN)_{19}$ isomers with less than four Ti–N bonds. These examples cover cases where the Ti atom is bound to a square, pentagon and hexagon face, with one, two and three Ti–N bonds formed. As we can see, all these structures are significantly higher in energy than the global minimum structure of Ti⊂$(BN)_{19}$, where the Ti atom is bound to a key hexagon with four Ti–N bonds formed.

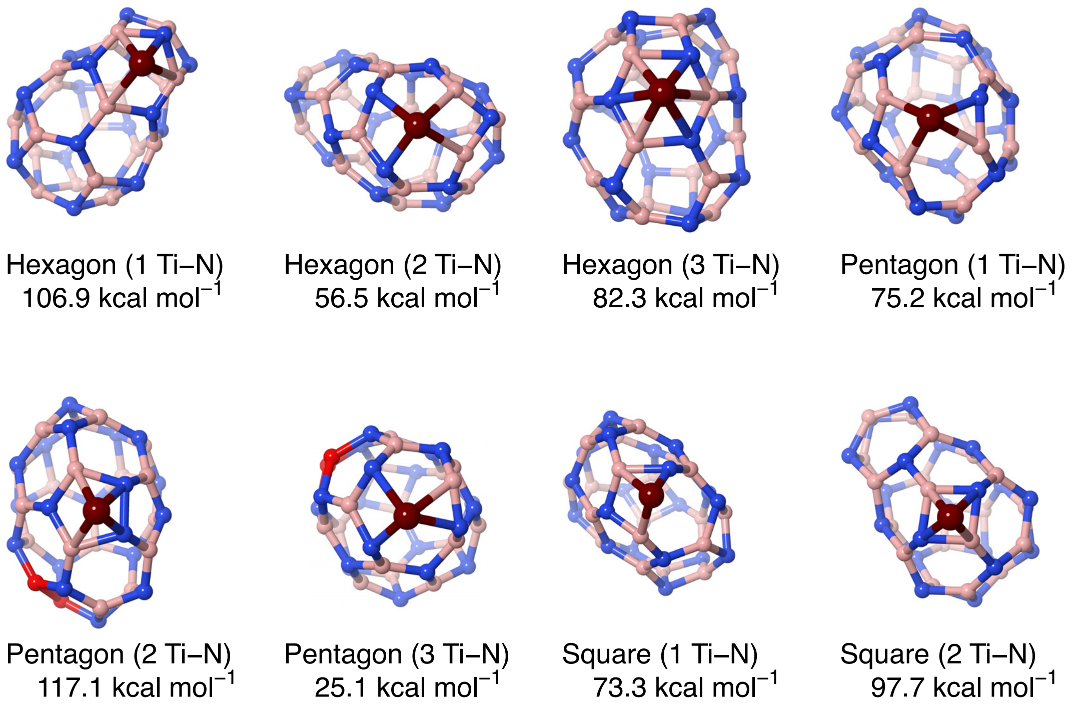

Supplementary Figure 5. Top view of molecular structures for Ti⊂$(BN)_{19}$ complexes having less than four Ti–N bonds. The polygonal face that hosts the Ti atom is indicated, and the number of formed Ti–N bonds is given in parentheses. Relative energies with respect to the global minimum are also provided, which were computed at the B3LYP-D3/6-31+G(d) level without including zero-point energy correction. Boron, nitrogen and titanium atoms are shown in pink, blue and dark red, respectively. B–B bonds are indicated by red color.

Supplementary Note 5: Binding the Ti atom with four N atoms of a heptagon or an octagon

Supplementary Figures 6 and 7 show the lowest-energy isomers among all possible complexes of Ti$\subset$(BN)$_n$ (n = 16, 19, 24) where the Ti atom is bound to a heptagon or an octagon forming four Ti–N bonds. As we can see, in all these isomers the four N atoms do not stay on the same plane (as shown by the side view structures), and the four Ti–N bonds are twisted compared to the global minimum structures where the N–Ti–N bond angles are more evenly distributed (see Supplementary Figures 11–13). As a result, all these structures are energetically less stable than the global minimum isomers.

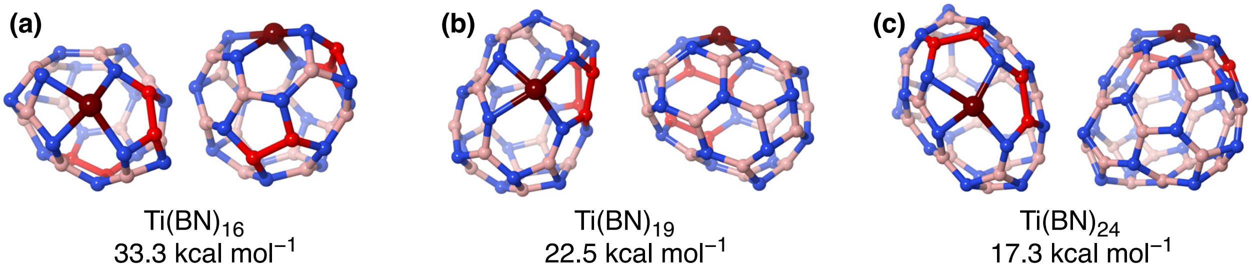

Supplementary Figure 6. Top view (left) and side view (right) of molecular structures for the lowest-energy Ti(BN)$_n$ isomers that have the Ti atom bound to a heptagonal face. (a) n = 16; (b) n = 19; (c) n = 24. B3LYP-D3/6-31+G(d) calculated relative energies (including zero-point energy correction) with respect to the global minimum are indicated. B, N and Ti atoms are shown in pink, blue and dark red, respectively. B–B bonds are indicated by red color.

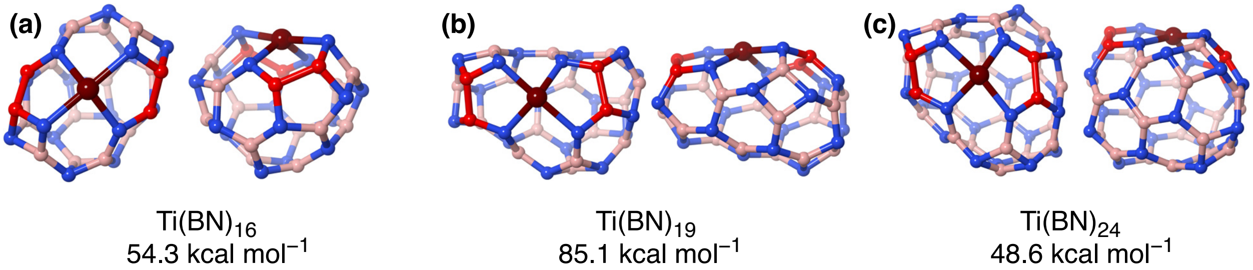

Supplementary Figure 7. Idem Supplementary Figure 6 for the lowest-energy Ti(BN)$_n$ isomers with an octagonal doping site.

Supplementary Note 6: Lowest-energy isomers of $Ti(BN)_n$

Here we present the 3D molecular structures and Schlegel diagrams of low-lying isomers of $Ti(BN)_n$ (n = 12, 13, 14, 16, 19 and 24). To be consistent with the notations used in the text, we denote cage **1** as the lowest-energy isomer of pristine cages $(BN)_n$, whereas cages **2**–**11** are the cage forms of the ten lowest-energy complexes $Ti\subset(BN)_n$ and are consecutively labeled according to the increasing energy of these complexes. For comparison, we also show the complex Ti⊂**1** and the endohedral complexes Ti@**1** and Ti@**2**.

The relative energies for all these isomers are summarized in Table 2 of the main text. The top view of 3D molecular structures and Schlegel diagrams of them are presented in Supplementary Figures 8–13.

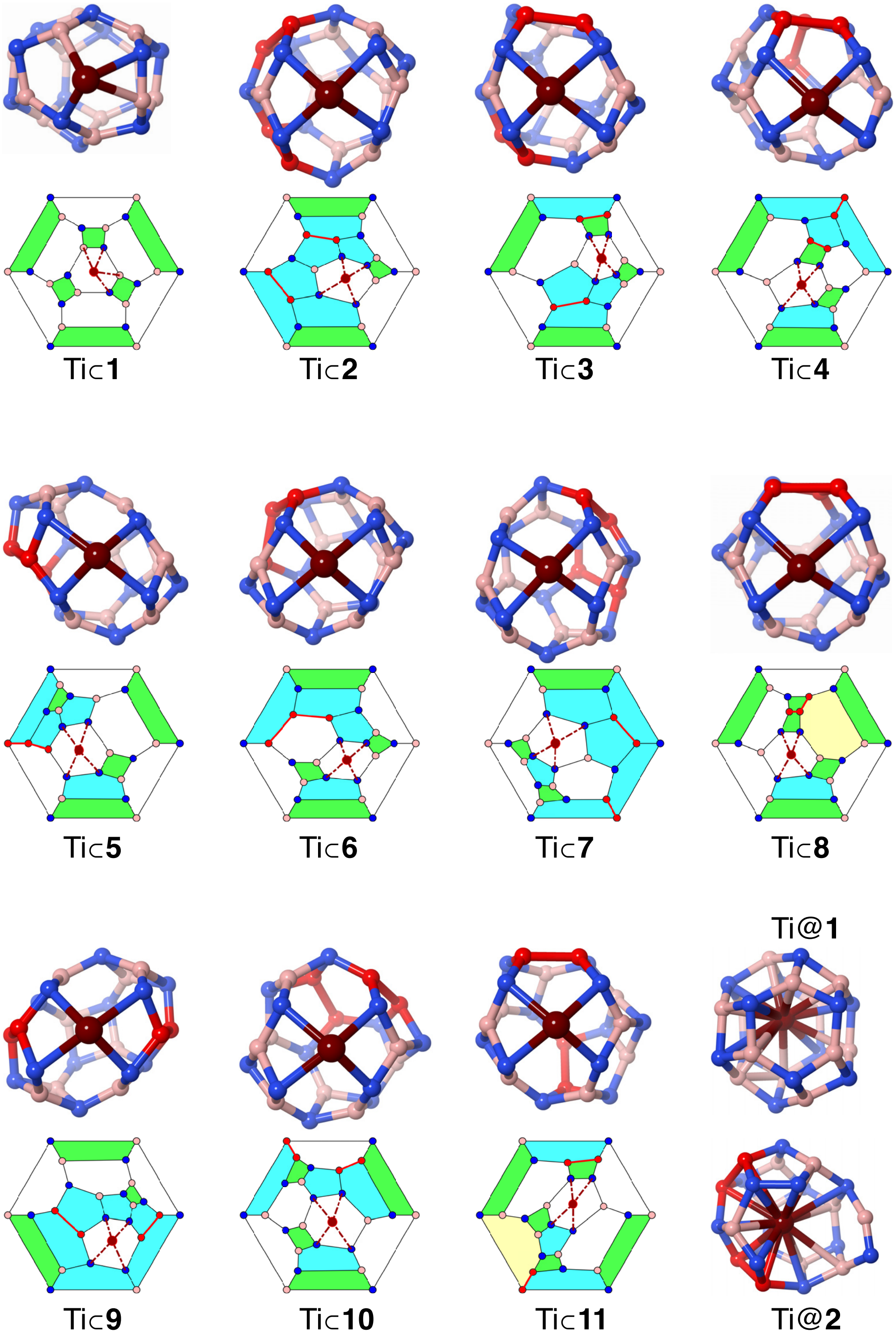

Supplementary Figure 8. Top view of molecular structures and Schlegel diagrams for Ti⊂$(BN)_{12}$ complexes, namely, Ti⊂**1**–Ti⊂**11**, as well as Ti@**1** and Ti@**2**. For the latter two endohedral species, only molecular structures are shown. Boron, nitrogen and titanium atoms are shown in pink, blue and dark red, respectively. B–B bonds are indicated by red color. In Schlegel diagrams, Ti–N bonds are indicated by dashed lines, and squares, pentagons, hexagons, heptagon and octagon are shown in green, cyan, white, yellow and magenta, respectively.

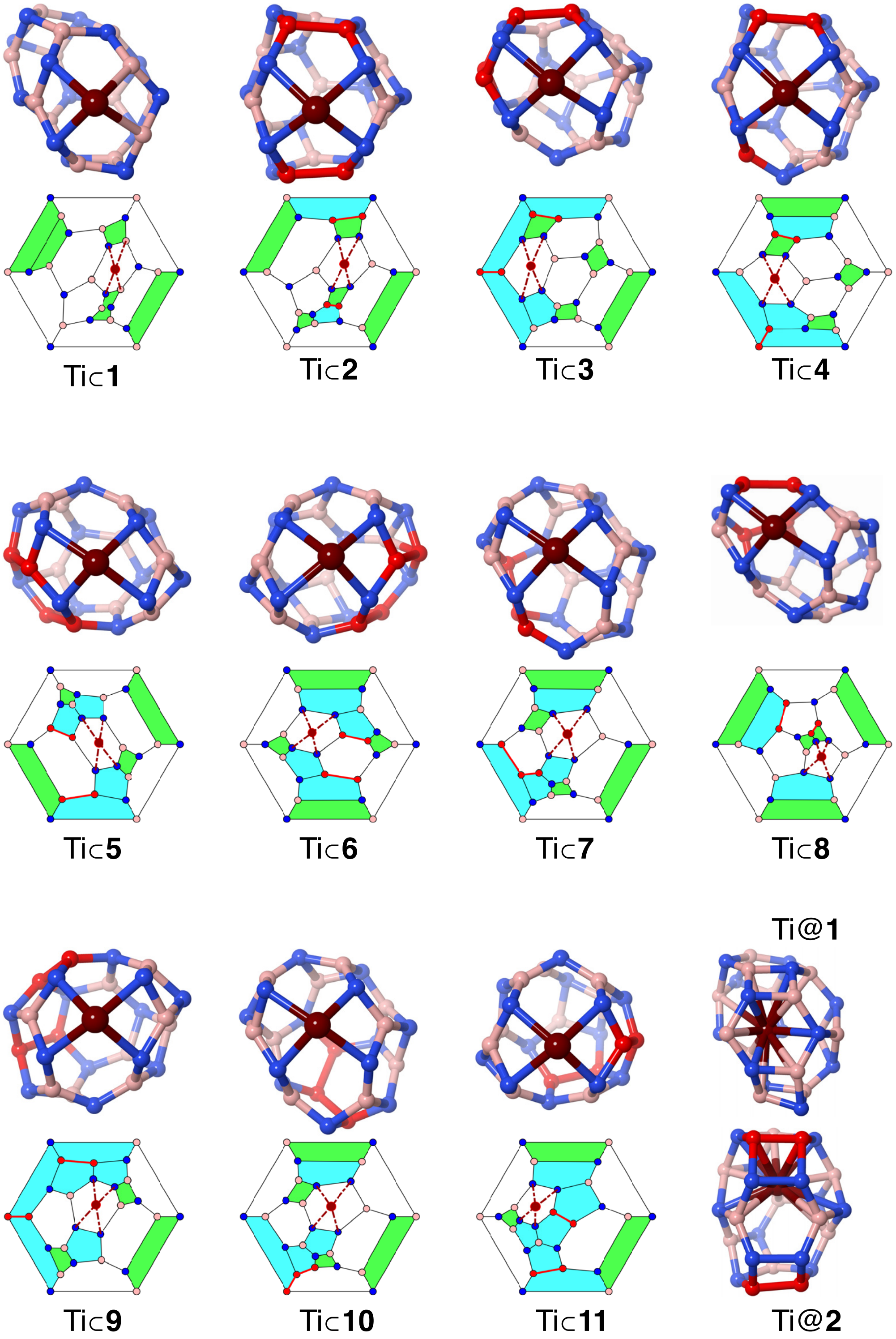

Supplementary Figure 9. Idem Supplementary Figure 8 for Ti$\subset$(BN)$_{13}$.

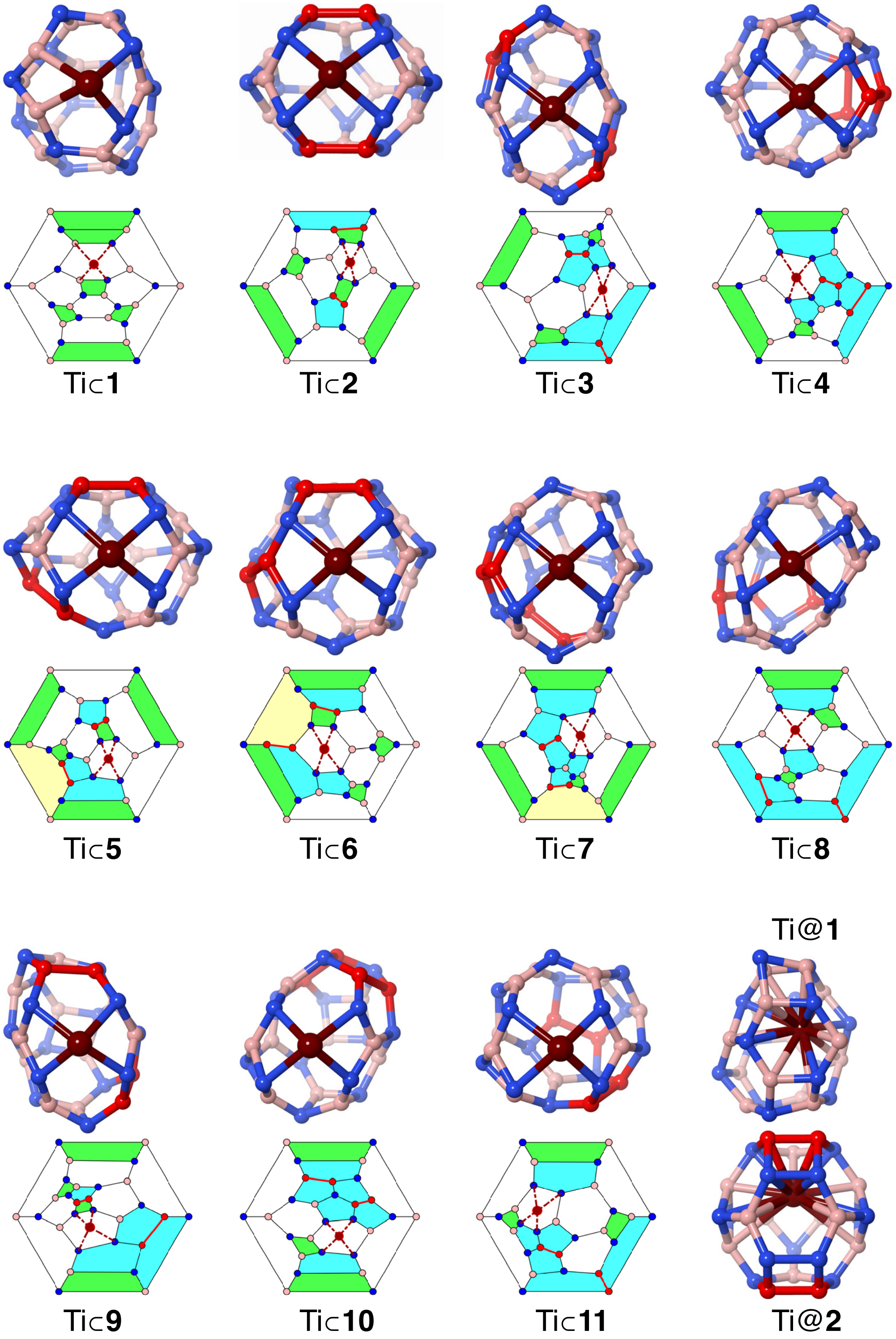

Supplementary Figure 10. Idem Supplementary Figure 8 for Ti⊂$(BN)_{14}$.

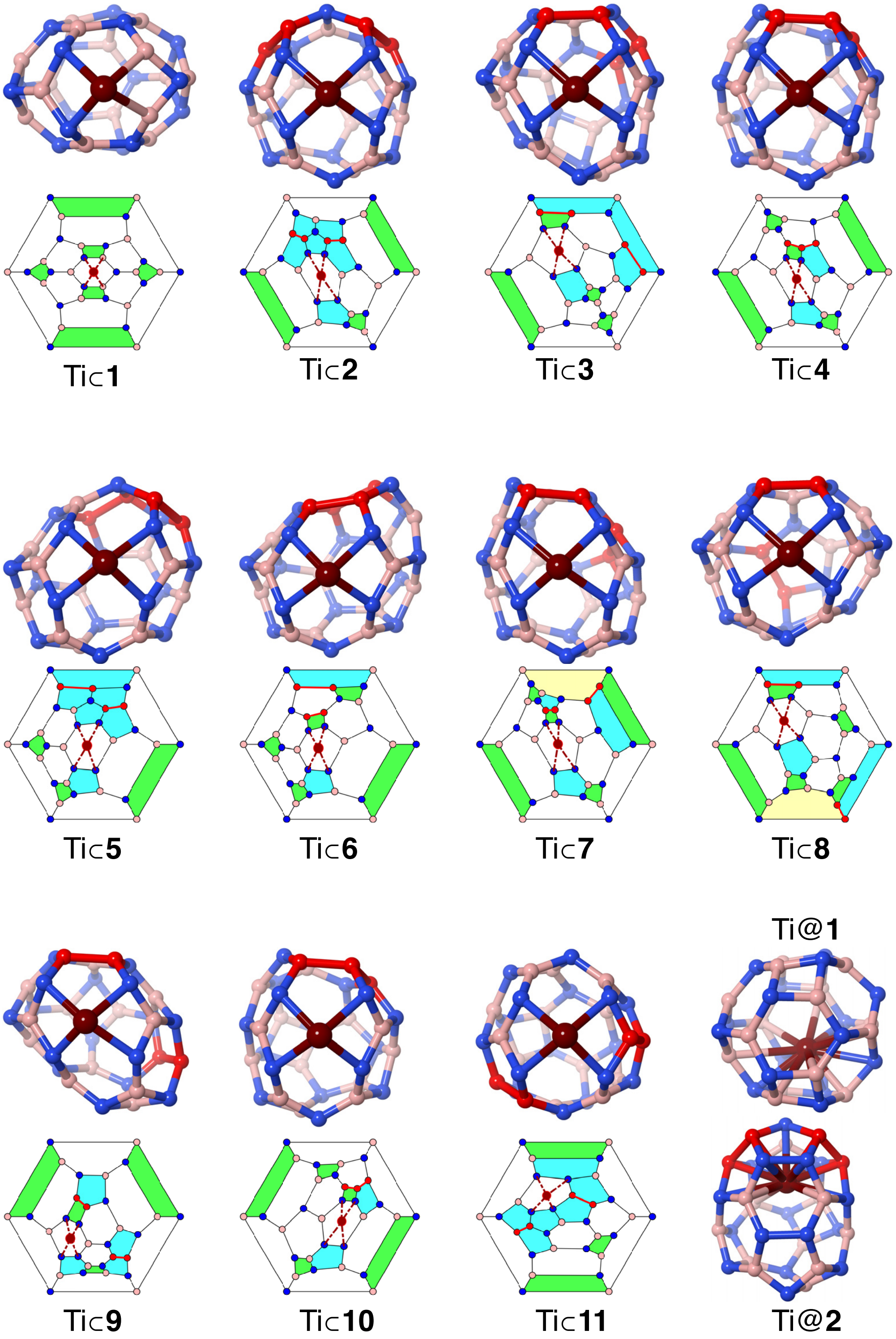

Supplementary Figure 11. Idem Supplementary Figure 8 for Ti⊂$(BN)_{16}$.

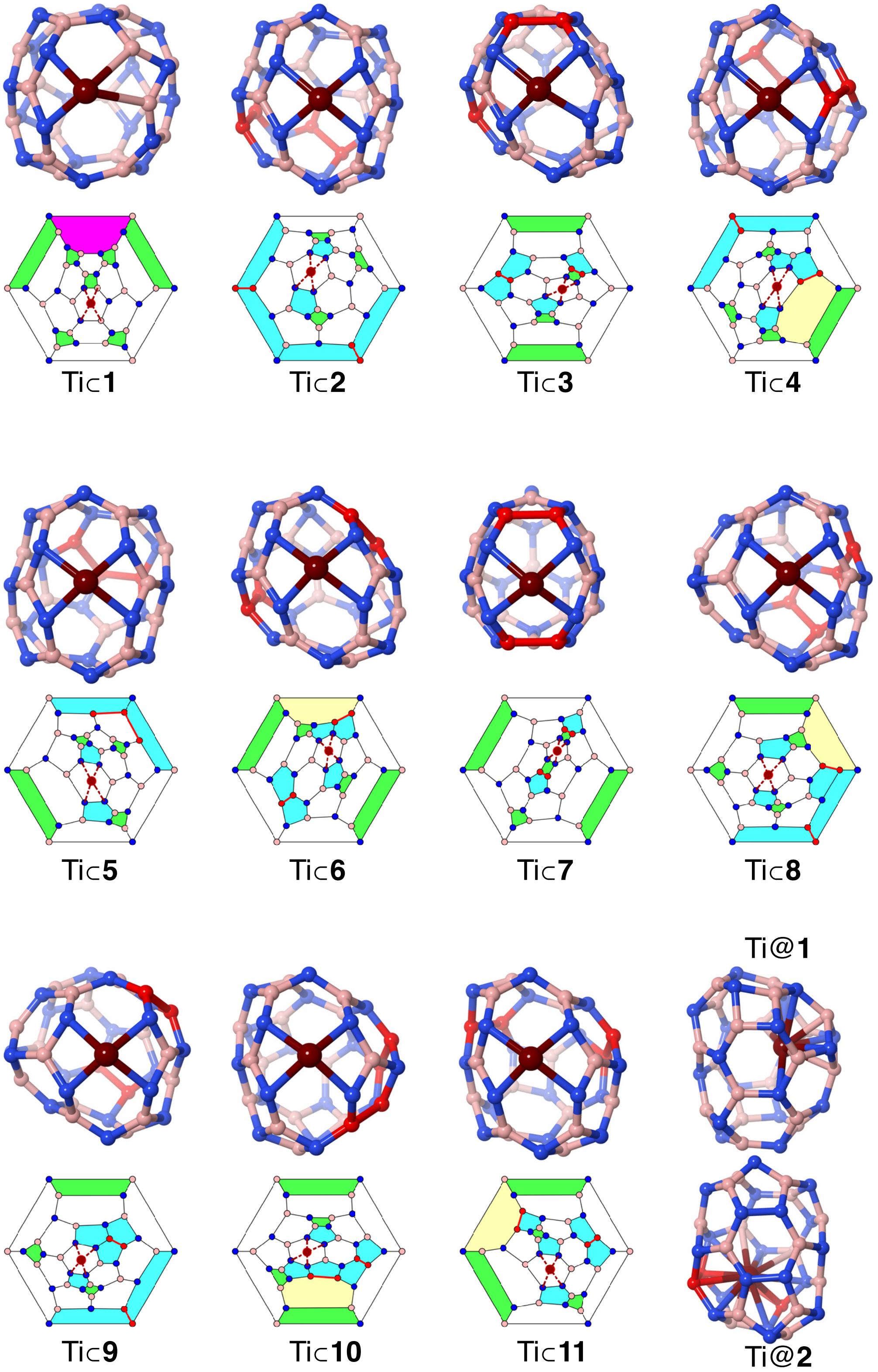

Supplementary Figure 12. Idem Supplementary Figure 8 for Ti$\subset$(BN)$_{19}$.

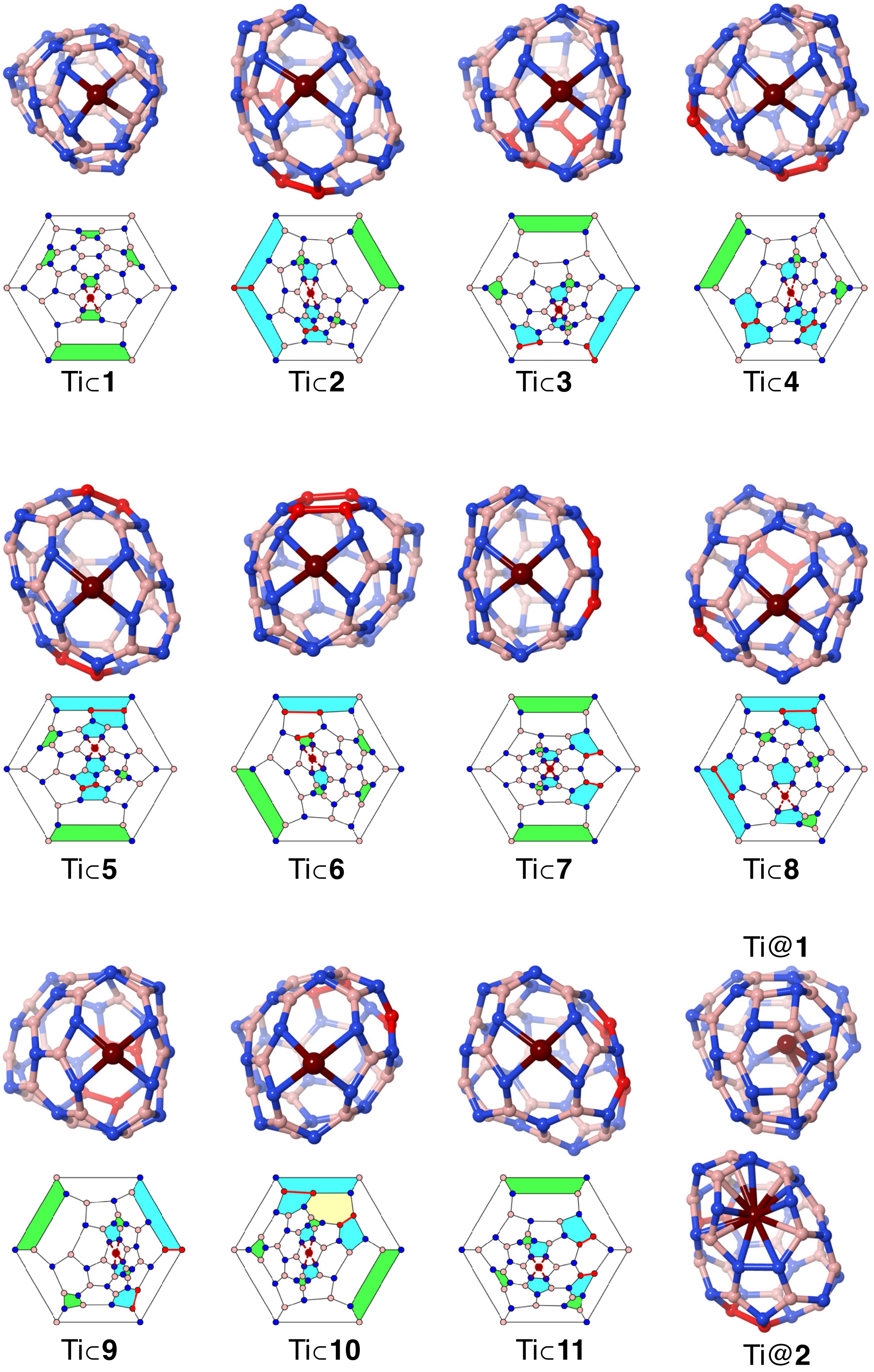

Supplementary Figure 13. Idem Supplementary Figure 8 for Ti⊂$(BN)_{24}$.

Supplementary Note 7: Lowest Gibbs free energy isomers of Ti(BN)$_n$

Supplementary Table 9 lists the lowest Gibbs free energy isomers at temperatures 0, 1500, 2000 and 2500 K for complexes Ti(BN)$_n$ and pristine cages (BN)$_n$ (n = 12, 13, 14, 16, 19 and 24). For the structures of the isomers of complexes, see Supplementary Note 6. As for the structures of the pristine cage isomers, Schlegel diagrams are presented in Supplementary Figures 14.

Supplementary Table 9. Lowest Gibbs free energy isomers at temperatures 0, 1500, 2000 and 2500 K and a pressure of 1 atm, for complexes Ti(BN)$_n$ (n = 12, 13, 14, 16, 19 and 24) and for pristine cages (BN)$_n$ (given in parentheses).

Temperature	$n = 12$	$n = 13$	$n = 14$	$n = 16$	$n = 19$	$n = 24$
0 K	Ti$\subset$**2** (**1**)	Ti$\subset$**2** (**1**)	Ti$\subset$**2** (**1**)	Ti$\subset$**2** (**1**)	Ti$\subset$**2** (**1**)	Ti$\subset$**2** (**1**)
1500 K	Ti$\subset$**8** (**1**)	Ti$\subset$**2** (**1a**)	Ti$\subset$**2** (**1**)	Ti$\subset$**2** (**1**)	Ti$\subset$**4** (**1**)	Ti$\subset$**3** (**1a**)
2000 K	Ti$\subset$**8** (**1**)	Ti$\subset$**2** (**1a**)	Ti$\subset$**2** (**1**)	Ti$\subset$**4** (**1**)	Ti$\subset$**4** (**1**)	Ti$\subset$**3** (**1a**)
2500 K	Ti$\subset$**8** (**1**)	Ti$\subset$**2** (**1a**)	Ti$\subset$**2** (**1**)	Ti$\subset$**4** (**1**)	Ti$\subset$**7** (**1**)	Ti$\subset$**3** (**1a**)

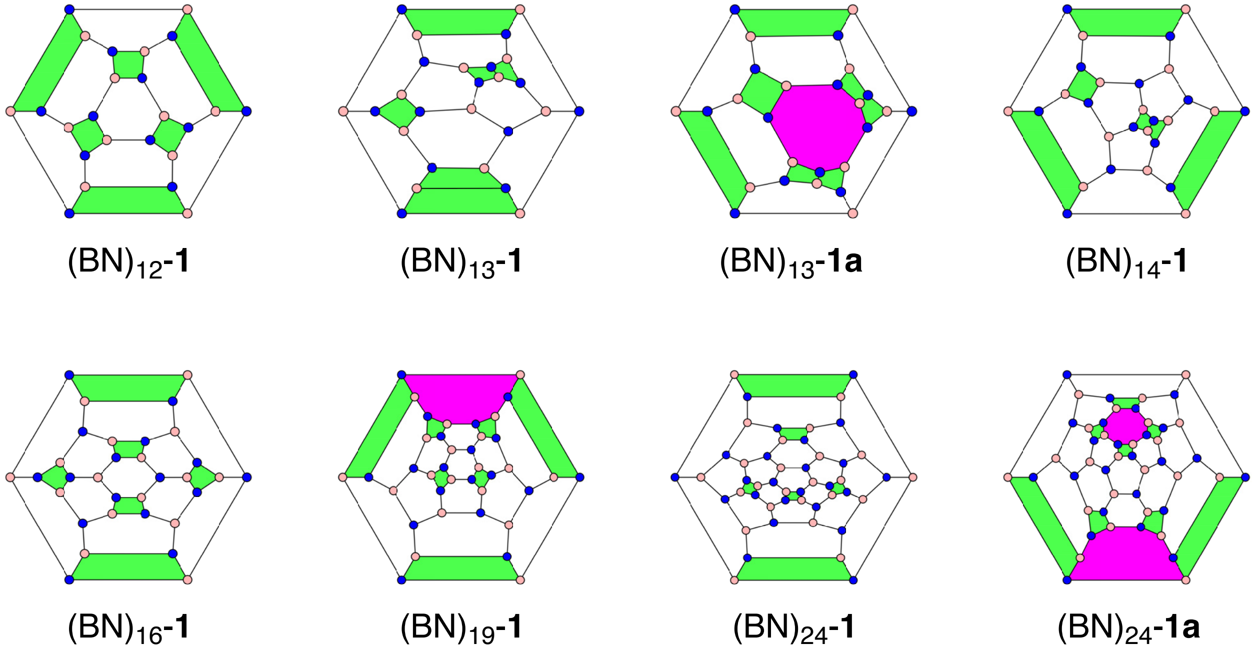

Supplementary Figure 14. Schlegel diagrams for the lowest Gibbs free energy isomers of pristine cages (BN)$_n$ (cf. Supplementary Table 9). Boron and nitrogen are shown in pink and blue, respectively. Squares, hexagons and octagon are shown in green, white and magenta, respectively.

Supplementary Note 8: Temperature-dependent mole fractions of major products of Ti(BN)$_n$

Assuming that the system has reached a thermodynamic equilibrium, temperature-dependent mole fractions are calculated on the basis of Maxwell–Boltzmann distribution.[10] Accordingly, the mole fraction of each species of m isomers is calculated as:

$$x_i = \frac{\sigma_i \exp[-G_i/(RT)]}{\sum_{j=1}^{m} \sigma_j \exp[-G_j/(RT)]} \tag{1}$$

where G_i is the Gibbs free energy of isomer i at temperature T, and σ_i is its symmetry number.[10] In the specific case of Ti$\subset$(BN)$_n$ clusters, σ_i is the number of symmetrically equivalent faces in the cage where the Ti atom is bound to. For instance, for an isomer containing a key hexagon, it is the number of equivalent hexagonal faces in the cage, multiplied by the number of equivalent ways of making a given hexagonal face a key hexagon. Moreover, if the isomer has an enantiomer, then σ_i is doubled. We have also combined the mole fractions of singlet and triplet states together, provided that the equilibrium structures of both states do not differ significantly and can be practically regarded as the same species.

Supplementary Figures 15–19 depict the temperature-dependent mole fractions for the major products of Ti(BN)$_n$ (n = 12, 13, 14, 16 and 24, respectively), at temperature range of 0–4000 K and a pressure of 1 atm. For comparison, relative concentration of isomer Ti$\subset$**1** is also shown, where **1** is the lowest-energy isomer of pristine cages. The distribution for $n = 19$ is already presented in Figure 5a in the text.

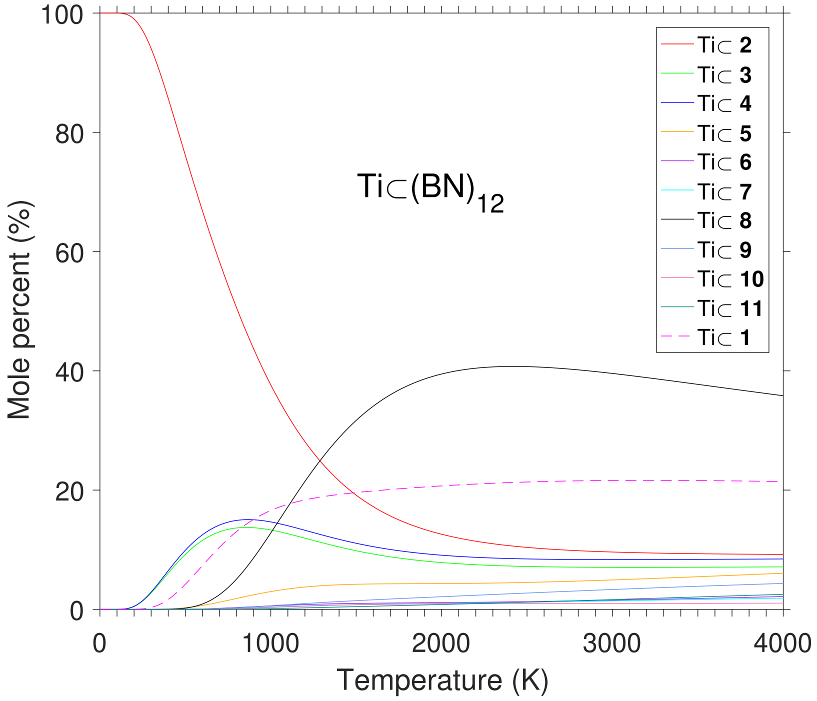

Supplementary Figure 15. B3LYP-D3/6-31+G(d) predicted mole fractions of the major products of Ti$\subset$(BN)$_{12}$, as a function of temperature.

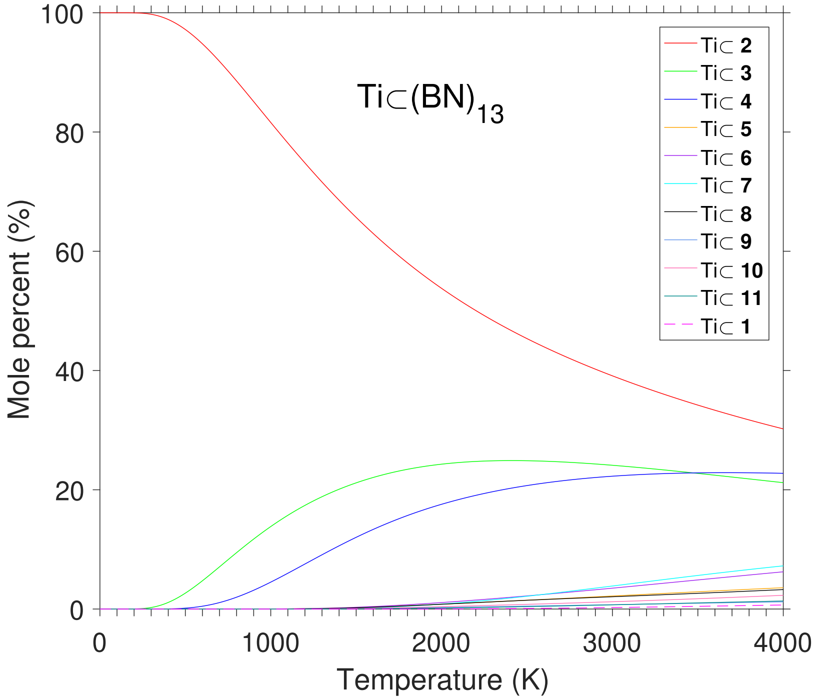

Supplementary Figure 16. Idem Supplementary Figure 15 for Ti$\subset$(BN)$_{13}$.

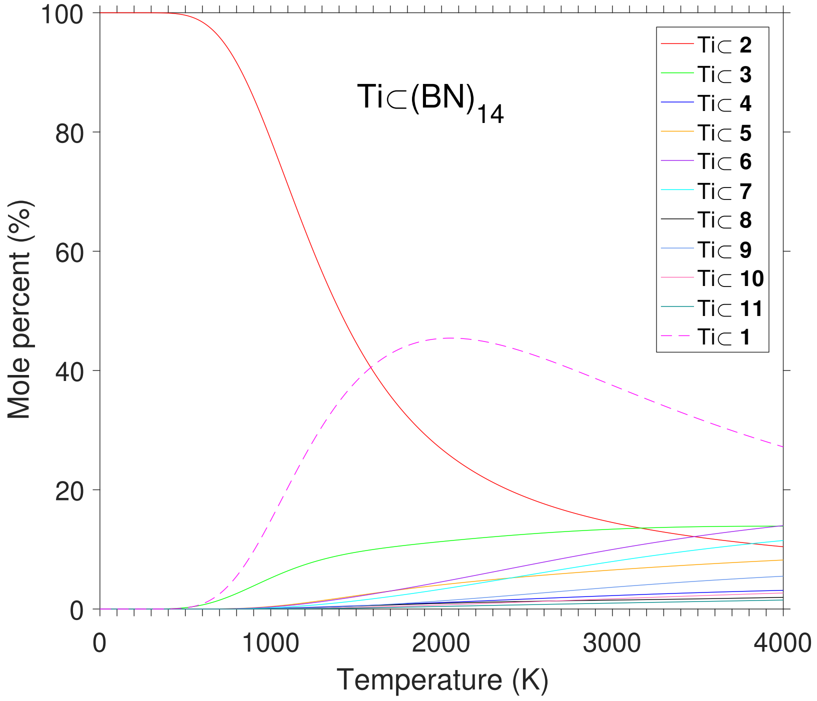

Supplementary Figure 17. Idem Supplementary Figure 15 for Ti$\subset$(BN)$_{14}$.

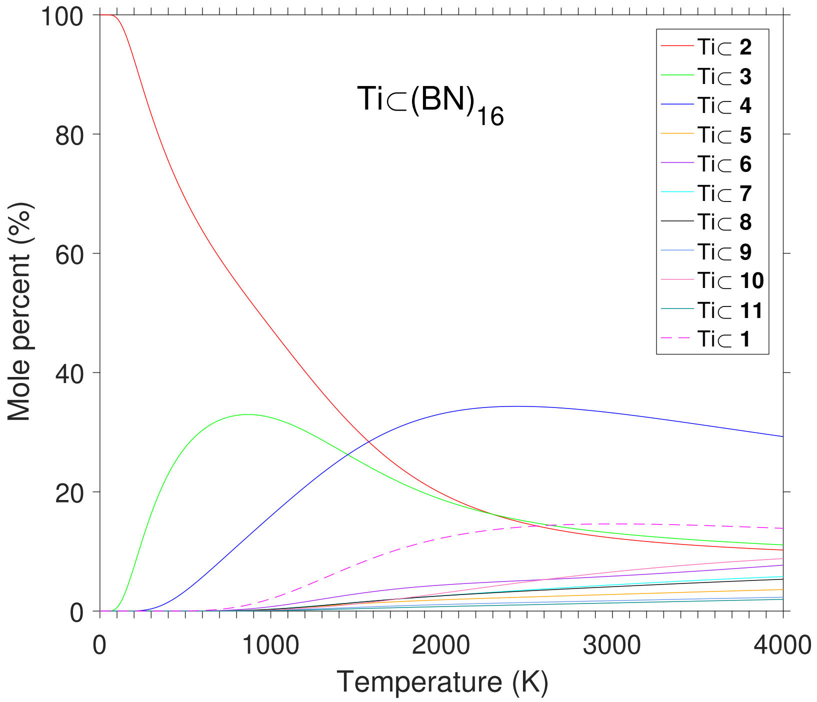

Supplementary Figure 18. Idem Supplementary Figure 15 for Ti⊂$(BN)_{16}$.

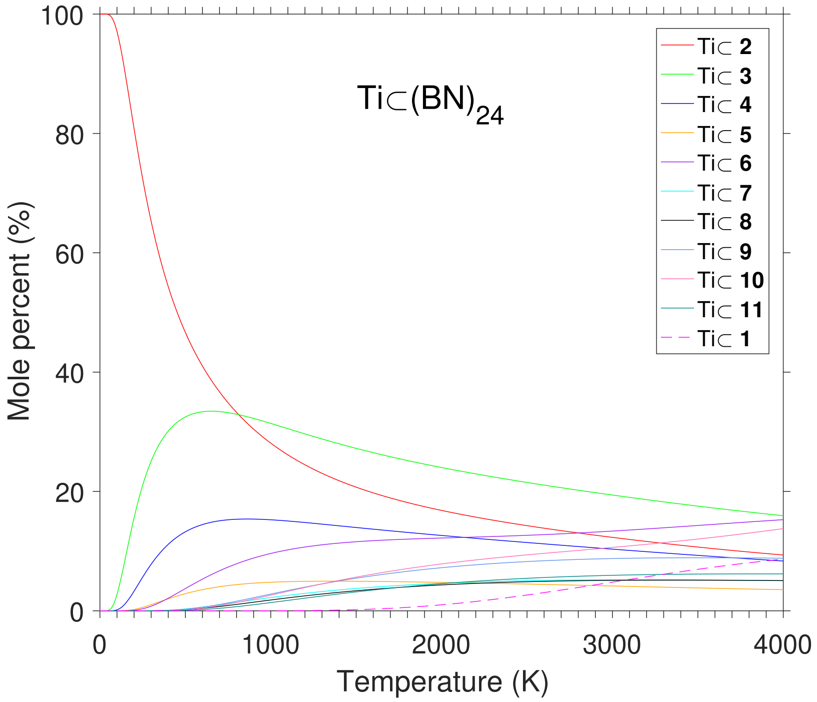

Supplementary Figure 19. Idem Supplementary Figure 15 for Ti⊂$(BN)_{24}$.

Supplementary References

(1) Brinkmann, G.; McKay, B. D. The program plantri, version 5.0. http://users.cecs.anu.edu.au/ bdm/plantri/, 2016.

(2) Fowler, P.; Manolopoulos, D. E. *An Atlas of Fullerenes*; Clarendon Press: Oxford, U.K., 1995.

(3) Wang, Y.; Díaz-Tendero, S.; Alcamí, M.; Martín, F. Topology-Based Approach to Predict Relative Stabilities of Charged and Functionalized Fullerenes. *J. Chem. Theory Comput.* **2018**, *14*, 1791–1810.

(4) Cormen, T.; Leiserson, C.; Rivest, R.; Stein, C. *Introduction to Algorithms*, 2nd ed.; MIT Press: Cambridge MA, 2001; Chapter 22.

(5) Fowler, P. W.; Horspool, D.; Myrvold, W. Vertex Spirals in Fullerenes and Their Implications for Nomenclature of Fullerene Derivatives. *Chem.: Eur. J.* **2007**, *13*, 2208–2217.

(6) Fowler, P. W.; Heine, T.; Mitchell, D.; Schmidt, R.; Seifert, G. Boron–nitrogen analogues of the fullerenes: the isolated-square rule. *J. Chem. Soc. Faraday Trans.* **1996**, *92*, 2197–2201.

(7) Grimme, S. Semiempirical hybrid density functional with perturbative second-order correlation. *J. Chem. Phys.* **2006**, *124*, 034108.

(8) Steinmetz, M.; Grimme, S. Benchmark study of the performance of density functional theory for bond activations with (ni,pd)-based transition-metal catalysts. *ChemistryOpen* **2013**, *2*, 115–124.

(9) Dohm, S.; Hansen, A.; Steinmetz, M.; Grimme, S.; Checinski, M. P. Comprehensive Thermochemical Benchmark Set of Realistic Closed-Shell Metal Organic Reactions. *J. Chem. Theory Comput.* **2018**, *14*, 2596–2608.

(10) Slanina, Z. Multimolecular clusters: Their isomerism and effective characteristics evaluated by quantum chemistry. *Int. J. Quantum Chem.* **1979**, *16*, 79–86.
