# Supplementary material for: Modification of boron nitride nanocages by titanium doping results unexpectedly in exohedral complexes
Source: Nat Commun. 2019 Oct 28;10:4908. doi: 10.1038/s41467-019-12877-0 (PMC6961409; doi:10.1038/s41467-019-12877-0)
Supplement: Supplementary file 3 — Description of Additional Supplementary Files [file 41467_2019_12877_MOESM3_ESM.docx]

**Description of Supplementary Files**

**File Name:** Supplementary Data 1

**Description:** Atomic coordinates of structure 1 for (BN)19

**File Name:** Supplementary Data 2

**Description:** Atomic coordinates of structure 2 for (BN)19

**File Name:** Supplementary Data 3

**Description:** Atomic coordinates of structure Ti⊂1 for (BN)19

**File Name:** Supplementary Data 4

**Description:** Atomic coordinates of structure Ti@1 for (BN)19

**File Name:** Supplementary Data 5

**Description:** Atomic coordinates of structure Ti⊂2 for (BN)19

**File Name:** Supplementary Data 6

**Description:** Atomic coordinates of structure Ti@2 for (BN)19

**File Name:** Supplementary Data 7

**Description:** Atomic coordinates of structure Ti⊂3 for (BN)19

**File Name:** Supplementary Data 8

**Description:** Atomic coordinates of structure Ti⊂4 for (BN)19

**File Name:** Supplementary Data 9

**Description:** Atomic coordinates of structure Ti⊂5 for (BN)19

**File Name:** Supplementary Data 10

**Description:** Atomic coordinates of structure Ti⊂6 for (BN)19

**File Name:** Supplementary Data 11

**Description:** Atomic coordinates of structure Ti⊂7 for (BN)19

**File Name:** Supplementary Data 12

**Description:** Atomic coordinates of structure 1 for (BN)12

**File Name:** Supplementary Data 13

**Description:** Atomic coordinates of structure 2 for (BN)12

**File Name:** Supplementary Data 14

**Description:** Atomic coordinates of structure Ti⊂1 for (BN)12

**File Name:** Supplementary Data 15

**Description:** Atomic coordinates of structure Ti@1 for (BN)12

**File Name:** Supplementary Data 16

**Description:** Atomic coordinates of structure Ti⊂2 for (BN)12

**File Name:** Supplementary Data 17

**Description:** Atomic coordinates of structure Ti@2 for (BN)12

**File Name:** Supplementary Data 18

**Description:** Atomic coordinates of structure 1 for (BN)13

**File Name:** Supplementary Data 19

**Description:** Atomic coordinates of structure 2 for (BN)13

**File Name:** Supplementary Data 20

**Description:** Atomic coordinates of structure Ti⊂1 for (BN)13

**File Name:** Supplementary Data 21

**Description:** Atomic coordinates of structure Ti@1 for (BN)13

**File Name:** Supplementary Data 22

**Description:** Atomic coordinates of structure Ti⊂2 for (BN)13

**File Name:** Supplementary Data 23

**Description:** Atomic coordinates of structure Ti@2 for (BN)13

**File Name:** Supplementary Data 24

**Description:** Atomic coordinates of structure 1 for (BN)14

**File Name:** Supplementary Data 25

**Description:** Atomic coordinates of structure 2 for (BN)14

**File Name:** Supplementary Data 26

**Description:** Atomic coordinates of structure Ti⊂1 for (BN)14

**File Name:** Supplementary Data 27

**Description:** Atomic coordinates of structure Ti@1 for (BN)14

**File Name:** Supplementary Data 28

**Description:** Atomic coordinates of structure Ti⊂2 for (BN)14

**File Name:** Supplementary Data 29

**Description:** Atomic coordinates of structure Ti@2 for (BN)14

**File Name:** Supplementary Data 30

**Description:** Atomic coordinates of structure 1 for (BN)16

**File Name:** Supplementary Data 31

**Description:** Atomic coordinates of structure 2 for (BN)16

**File Name:** Supplementary Data 32

**Description:** Atomic coordinates of structure Ti⊂1 for (BN)16

**File Name:** Supplementary Data 33

**Description:** Atomic coordinates of structure Ti@1 for (BN)16

**File Name:** Supplementary Data 34

**Description:** Atomic coordinates of structure Ti⊂2 for (BN)16

**File Name:** Supplementary Data 35

**Description:** Atomic coordinates of structure Ti@2 for (BN)16

**File Name:** Supplementary Data 36

**Description:** Atomic coordinates of structure 1 for (BN)24

**File Name:** Supplementary Data 37

**Description:** Atomic coordinates of structure 2 for (BN)24

**File Name:** Supplementary Data 38

**Description:** Atomic coordinates of structure Ti⊂1 for (BN)24

**File Name:** Supplementary Data 39

**Description:** Atomic coordinates of structure Ti@1 for (BN)24

**File Name:** Supplementary Data 40

**Description:** Atomic coordinates of structure Ti⊂2 for (BN)24

**File Name:** Supplementary Data 41

**Description:** Atomic coordinates of structure Ti@2 for (BN)24
