# Supplementary material for: Modification of boron nitride nanocages by titanium doping results unexpectedly in exohedral complexes
Source: Nat Commun. 2019 Oct 28;10:4908. doi: 10.1038/s41467-019-12877-0 (PMC6961409; doi:10.1038/s41467-019-12877-0)
Supplement: Supplementary file 2 — Peer Review File [file 41467_2019_12877_MOESM2_ESM.pdf]

REVIEWERS' COMMENTS:

Reviewer#1

Review of “Unexpected modification of boron nitride nanocages by titanium doping: exohedral complexes prevail over endohedral ones”

Authors: Li, Wang

This manuscript is worthy of publication in Nature Communications, but the authors should address the following issues before publication:

1) Ti coordination to the nitrogen appears to break N-N bond in the cage. Why is the bond-breaking necessary? Couldn’t the Ti atom coordinate to the nitrogen lone pair without breaking any bonds?

2) Is the behavior of Ti towards the BN nanocages a function of being an early transition metal? Would a late transition metal (e.g. iron, nickel, zinc) behave differently?

3) Does this journal prefer kcal/mole or kJ/mole energies?

4) Why is the Methods section placed so late in the manuscript?

5) The DFT calculations were carried out with the 6-31+G(d) basis set, which includes diffuse functions. Were any calculations carried out without the diffuse functions to determine how necessary the diffuse functions are?

Douglas Strout

Reviewer#2

In this work, the authors carried out detailed DFT computations and demonstrated that Ti(BN)n (n = 12–24) complexes have an exohedral structure instead of an endohedral one. However, the conclusion is not that kind of unexpected. Basically this is a well performed theoretical work, and the manuscript is also clearly written. However, it is in lack of enough novelty for publication in NC.

In the community, it is well known that the preference of endohedral or exohedral complexes of such cage structures depends on the cage size, the charge transfer between the endospecies and the cages, and the resulting interactions between them, among others.

Even for carbon cages, it is not always true that the endohedral fullerenes are preferred. For example, in the paper titled Is Fullerene C60 Large Enough to Host a Multiply Bonded Dimetal?, published in J. AM. CHEM. SOC. 2008, 130, 7459–7465, it showed that “some dimetal fullerenes M2@C60 (M= Cr, Mo, W) have been studied with computational quantum chemistry methods. The transition metal diatomic molecules Cr2, Mo2, W2 form exohedral complexes with C60”.

Reviewer#3

Nature Communications

Review of

“Unexpected modification of boron nitride nanocages by titanium doping: exohedral complexes prevail over endohedral ones”

The manuscript entitled, “Unexpected modification of boron nitride nanocages by titanium doping: exohedral complexes prevail over endohedral ones”, authored by R. Li and Y. Wang investigates the relative stability of various symmetry conformers of Ti(BN)n (n = 12-24) nanocages using the DFT-B3LYP-D3 theoretical model with various basis sets. Single-point calculations were carried with the double-hyrbrd B2PLYP-D3/Def2-TZVP. The authors report the theoretical energetic stability of the Ti(BN)n nanocages in relation to the exohedral and endohedral conformers. Additionally, the authors provide insight into the respective electronic and thermodynamic characteristics of the exohedral complexes.

General Evaluation:

The authors report on a topic that is of considerable interest to theoretical as well as experimental chemists. Although some revision is requested (see below), the overall manuscript is of incredibly high quality, including a very thorough and impressive description of the systematic approach applied (as described in the supplemental information) to this research investigation. After addressing the below concerns, the manuscript warrants publication.

Minor Suggested Modifications (primarily grammatical in nature)

Second Paragraph

“intruiging” is misspelled.

Fourth Paragraph

“The doped cage contains also a few pentagons…” delete “also”

Major Suggested Modifications

1. It may be due to the format prescribed by Nature Communications, but I found it frustrating not having tabulated data embedded in the text. Referring to the Supplement Information (SI) was cumbersome and took away from the quality of the manuscript. If able to do so, please provide truncated tables in the text to further substantiate your findings.

2. I would like to see a formal description in the manuscript for the meaning behind the ‘variable’ [illegible] used in Figure 4. In print format, it may be easier to decipher what [illegible] represents, but a thorough discussion in the text would help clarify any confusion.

3. What is the citation for the claim that “… observed cage structure of many endohedral carbon metallofullerenes differs dramatically…”? The distortion that arises for small fullerenes comes in the form of a slight increase in the total volume of the system with some potential covalent bonding between the metal and the carbon cage, and not a “dramatic” change. This statement needs to be either omitted or citations provided to substantiate this statement.

4. The claim that “… surprising result since it is well-known that carbon metallofullerenes are generally endohedral…” is primarily the case with large fullerenes. Reference #21 does not address

metallofullerenes smaller than C60 cages, although Reference #26 does provide a few examples of endohedral C28 complexes. For small cage structures with a high degree of curvature, the exohedral positioning of the metal is predominantly favored. See Comput. Theor. Chem. 2017, 1119, 32-44 and Int J. Quantum Chem. 2019, e25992 as examples. In general, the exohedrally favored position of the Ti is not necessarily startling for small cages with high curvature. What is interesting is the bonding characteristics of the Ti ion… bonded to 4 N atoms rather than just a single N atom.

Answer to the referees

We thank very much all reviewers for their time and efforts in reviewing our manuscript. We are especially grateful to Reviewers #1 and #3 for their appreciation of this work and for their valuable comments. After carefully considering the reviewers questions and suggestions, we made necessary modifications in the manuscript and the Supporting Information. We also performed some additional calculations, with which we hope that question 2 of Reviewer #1 can be satisfactorily answered.

In the following we provide a detailed answer to reviewers' questions and describe all changes introduced in the revised version of the paper. All changes in the manuscript are indicated in red in its marked-up version, provided as a separate file.

A point-by-point response to the reviewers' questions are presented in the following pages (with their comments indicated in bold italics).

Reviewer#1

This manuscript is worthy of publication in Nature Communications, but the authors should address the following issues before publication:

1) Ti coordination to the nitrogen appears to break N-N bond in the cage. Why is the bond-breaking necessary? Couldn't the Ti atom coordinate to the nitrogen lone pair without breaking any bonds?

We think that the Ti atom cannot stably coordinate to the nitrogen lone pair without breaking any N–N bonds. The reason is as follows.

In a pristine BN cage containing N–N bonds, the lone pairs of N atoms correspond to N's doubly occupied p_z orbitals, participating in the delocalized π bonding with B atoms (as shown in Figure 1). It is known that the system is significantly stabilized by the donor-acceptor interaction with electron transfer from nitrogens' lone pairs to borons' empty p_z orbtials (see the curly cyan arrows in Figure 1).

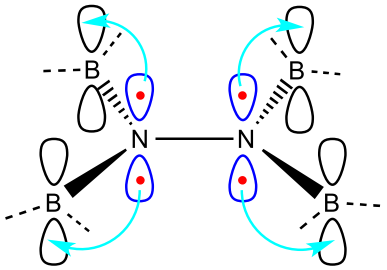

Figure 1. *Schematic illustration of the delocalized π bonding in a pristine BN cage containing N–N bonds. Occupied π orbitals are depicted in blue and electrons originated from nitrogen are in red. Curly cyan arrows indicate the flow of electrons in the donor-acceptor interaction from nitrogens' occupied p_z orbitals to borons' empty p_z orbitals.*

When bonding to a Ti atom, if the N–N bond is not broken, each N atom becomes tetracoordinated and thus its valence orbitals undergo from sp^2 to sp^3 hybridization, as illustrated in Figure 2. As the Ti gives away all its four valence electrons (indicated by green crosses), each N ligand would get one of them. Consequently, an electron from each N's lone pair must go to the empty p_z orbital of either of the two neighboring B atoms, since each N atom is already saturated with eight electrons in its valence shell (see Figure 2). This bonding situation is not energetically favorable for three reasons: (i) The delocalized π bonding between N and its neighboring B atoms is destroyed, as the lone pair of each N forms a coordination bond with the Ti^{4+} and thus no longer participates in the π system. (ii) As a consequence of (i), one of the two neighboring B atoms gets a π electron in its p_z orbital and thus takes a formal charge of -1 (which is not a favorable situation for the less electronegative boron), while the other B atom has an empty p_z orbital without receiving any electron donation from N. (iii) The conformation of these sp^3 N atoms in this bonding situation is energetically unfavorable. Considering that the typical Ti–N bond length is ~2.0 Å and the N–N bond length is ~1.5 Å, the Ti–N–N angle would be ~40 degree (see Figure 2), which is too small for an ideal bond angle of sp^3 hybridization (~109.5 degree). All these factors suggest that the Ti cannot stably coordinate to N without breaking any N–N bond.

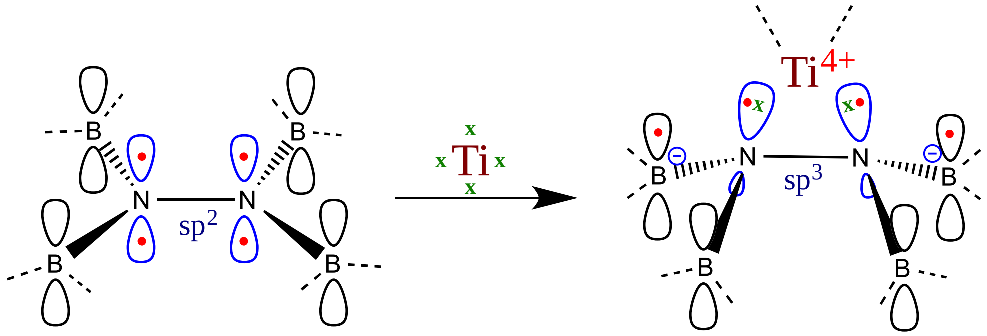

Figure 2. *Forming Ti–N coordination bonds without breaking N–N bond. Upon coordinating to the Ti atom, the orbital hybridization of each N atom changes from sp^2 to sp^3. Meanwhile, each N atom gains one electron from Ti (indicated by a green cross), and thus an electron from its lone pair (indicated by a red dot) goes to the empty p_z orbital of either the two neighboring B atoms. Note that only two N ligands are explicitly drawn and the other two Ti–N bonds are indicated by dashed lines for simplicity.*

By contrast, by breaking N–N bonds, N atoms can stably coordinate to the Ti atom while maintaining the delocalized π bonding between N and B atoms as in the pristine cage, as shown in Figure 3. This is because the cleavage of N–N bond leaves each N atom a free valence electron (indicated by a red dot), which combines with another electron taken from the Ti (indicated by a green cross), offering each N atom a second lone pair (indicated in magenta). It is this lone pair that allows each N atom to stably coordinate to the Ti^{4+} (see Figure 3). In this way, the complex is stabilized by forming four N→Ti^{4+} bonds without destroying the π system between N and B, only at expense of breaking a relatively week N–N bonds.

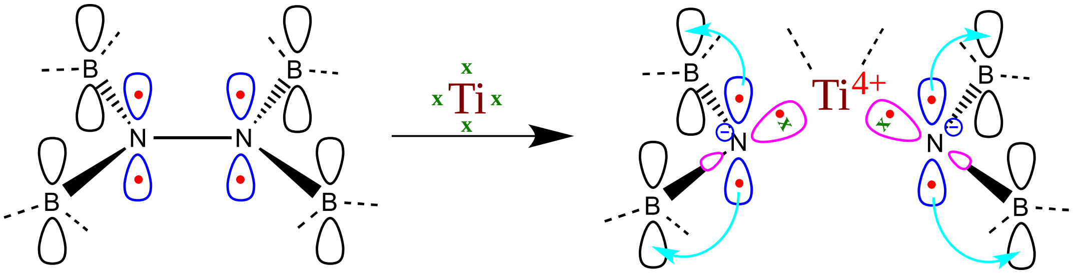

Figure 3. *Forming Ti–N coordination bonds by breaking N–N bonds. The second lone pair is indicated in magenta color.*

Based on the above discussions, we have added a sentence in the revised version of the manuscript (page 6) to briefly clarify this issue.

2) Is the behavior of Ti towards the BN nanocages a function of being an early transition metal? Would a late transition metal (e.g. iron, nickel, zinc) behave differently?

These are very interesting questions. To give a sensible answer to them, we have conducted a series of calculations, though preliminary, for $(BN)_{19}$ nanocages doped with a single atom of transition metals from Sc to Zn. We have also considered Zr, which belongs to the same fourth group as Ti. In brief, the answer to the reviewer's questions is yes: the behavior of Ti towards the BN nanocages is probably owing to

the higher bonding ability (valency) of early transition metals; and indeed, late transition metals behave differently in this respect.

For each transition metal M (M = Sc, Ti, V, …, Zn and Zr), we have considered three isomers of $M(BN)_{19}$, the exohedral M⊂**2** and the endohedral M@**1**, M@**2** (following the same notations used in the manuscript: the cage form **2** is the one in the global minimum structure of $Ti(BN)_{19}$, while cage form **1** corresponds to the lowest-energy isomer of pristine $(BN)_{19}$ cages). All structures were fully optimized at the B3LYP-D3BJ/6-31G(d) level. The ground state was determined by taking into account both singlet and triplet states (for systems containing an even number of electrons) or both doublet and quartet states (for systems containing an odd number of electrons), and confirmed by wavefunction stability tests.

Figure 4 plots the relative energies between the three $M(BN)_{19}$ isomers. As we can see, the exohedral structure (M⊂**2**, full red circle) has the lowest energy for early transition metals up to Fe. For Co, the endohedral structure (M@**1**, empty blue triangle) lies slightly lower in energy (by ~3.5 kcal/mol) than the exohedral one (M⊂**2**, full red circle). Beyond Co, the endohedral M@**1** is significantly more stable than the exohedral M⊂**2**, by ~30–50 kcal/mol. These results clearly demonstrate that late transition metals indeed behave differently from early transition metals in modifying the structure of BN nanocages. The reason for this is probably that early transition metals have a greater of number of available valence electrons (viz., valency) than late transition metals. For instance, Ti has four available valence electrons, allowing it to fully coordinate to the four N atoms. In comparison, Ni and Zn normally have only two available valence electrons, since their typical oxidation state is +2. Consequently, Ni or Zn can only coordinate to maximally two of the four N atoms in cage **2**, making isomer M⊂**2** much less stable than isomer M@**1**. This interpretation is supported by the sum of Wiberg bond indices of the metal, which is 3.3 and 0.9 for Ti⊂**2** and Zn⊂**2**, respectively, indicating that Ti exhibits much higher bonding ability (or valency) than Zn in the complex. Additionally, Zr shows similar result to Ti (see Figure 4), implying that the behavior of the metal is mainly determined by its valency.

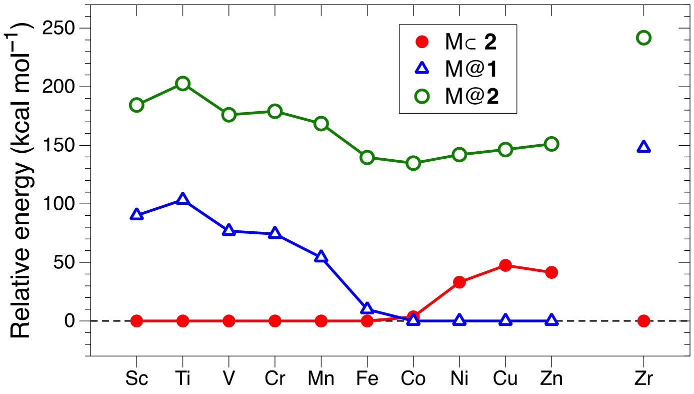

Figure 4. *Relative energy (including zero-point energy correction) between the three $M(BN)_{19}$ isomers, M⊂**2**, M@**1** and M@**2** (M = Sc, Ti, …, Zn and Zr). Full red circles represent the exohedral isomer M⊂**2**. Empty blue triangles and empty green circles indicate the endohedral isomers M@**1** and M@**2**, respectively.*

We are aware that the above preliminary study on the behavior of different transition metals is far from been complete. As the valency (or oxidation state) varies with the

metal, the bonding patterns in the low-lying structures of M(BN)n complexes would likely change from metal to metal, and thus might be distinct from those for Ti(BN)n as presented in the manuscript. We think that an in-depth study on this issue goes beyond the scope of the present paper and should be the object of future work. According to the above discussions, in the revised version of the manuscript (page 13), we have amended the first sentence in the last paragraph of the Results section and indicate that different transition metals may show different behaviors in modifying the structures of BN nanocages, which requires systematic studies in the future.

3) Does this journal prefer kcal/mole or kJ/mole energies?

We preferred to use kcal/mole for it is still commonly used in the community. However, if the referee and/or the editor think that the use of kJ/mole is more appropriate, we would be happy to take that option.

4) Why is the Methods section placed so late in the manuscript?

We did so because we wanted to place all technical details at the end, in order not to distract the readers from the main findings of this work.

5) The DFT calculations were carried out with the 6-31+G(d) basis set, which includes diffuse functions. Were any calculations carried out without the diffuse functions to determine how necessary the diffuse functions are?

We did perform some calculations using basis set without diffuse functions, namely, 6-31G(d). As shown in Supplementary Figures 3 and 4, the basis sets 6-31G(d) and 6-31+G(d) give very similar relative isomer energies for both $Ti(BN)_{19}$ and $Ti(BN)_{24}$ systems. Hence, the inclusion of diffuse functions in the basis set is not necessary in the present study. For this reason, we employed the 6-31G(d) basis set in the calculations for prescreening of low energy isomer candidates, as mentioned in the Methods section of the manuscript.

Reviewer#3

The manuscript entitled, "Unexpected modification of boron nitride nanocages by titanium doping: exohedral complexes prevail over endohedral ones", authored by R. Li and Y. Wang investigates the relative stability of various symmetry conformers of Ti(BN)n (n = 12-24) nanocages using the DFT-B3LYP-D3 theoretical model with various basis sets. Single-point calculations were carried with the double-hyrbrd B2PLYP-D3/Def2-TZVP. The authors report the theoretical energetic stability of the Ti(BN)n nanocages in relation to the exohedral and endohedral conformers. Additionally, the authors provide insight into the respective electronic and thermodynamic characteristics of the exohedral complexes.

General Evaluation:

The authors report on a topic that is of considerable interest to theoretical as well as experimental chemists. Although some revision is requested (see below), the overall manuscript is of incredibly high quality, including a very thorough and impressive description of the systematic approach applied (as described in the supplemental information) to this research investigation. After addressing the below concerns, the manuscript warrants publication.

We thank the reviewer for his/her high appreciation of our work.

Minor Suggested Modifications (primarily grammatical in nature)

Second Paragraph
"intruiging" is misspelled.

Corrected.

Fourth Paragraph
"The doped cage contains also a few pentagons…" delete "also"

We have removed it in the revised version of the manuscript.

Major Suggested Modifications

1. It may be due to the format prescribed by Nature Communications, but I found it frustrating not having tabulated data embedded in the text. Referring to the Supplement Information (SI) was cumbersome and took away from the quality of the manuscript. If able to do so, please provide truncated tables in the text to further substantiate your findings.

We followed the reviewer's advice. We have truncated Supplementary Table 6 in the original version of Supplementary Information and placed it in the revised manuscript (as Table 1). We have kept the original Table in the revised Supplementary

Information in order to provide more examples to support our findings. Moreover, we moved Supplementary Table 9 from the original version of the Supplementary Information to the revised manuscript (as Table 2). The corresponding text has been adapted for these changes in the revised manuscript (page 7 for Table 1; pages 9 and 10 for Table 2).

2. I would like to see a formal description in the manuscript for the meaning behind the ‘variable’ □ used in Figure 4. In print format, it may be easier to decipher what □ represents, but a thorough discussion in the text would help clarify any confusion.

We suppose that the reviewer referred to the ‘variable’ lambda used in Figure 4 (the letter ‘□’ displayed in this comment is unreadable). Following this suggestion, we have explicitly explained the meaning of the variable lambda in the legend of Figure 4, and added informative text to the discussion referring to the figure (page 8).

3. What is the citation for the claim that “… observed cage structure of many endohedral carbon metallofullerenes differs dramatically…”? The distortion that arises for small fullerenes comes in the form of a slight increase in the total volume of the system with some potential covalent bonding between the metal and the carbon cage, and not a “dramatic” change. This statement needs to be either omitted or citations provided to substantiate this statement.

In that sentence, what we actually wanted to say was that the change of the topology of cage framework (viz., the connectivity between atoms), not just the distortion of cage structure (which retains the cage topology), is observed upon encapsulation of the metal. We thank the reviewer for pointing out the ambiguity of that sentence. We have rephrased it in the revised manuscript (page 3), as “More generally, the observed cage topology (viz., the connectivity between atoms) of many endohedral carbon metallofullerenes differs dramatically from that of the corresponding neutral empty fullerenes”. We also provided the corresponding citations to that statement.

4. The claim that “… surprising result since it is well-known that carbon metallofullerenes are generally endohedral…” is primarily the case with large fullerenes. Reference #21 does not address metallofullerenes smaller than C60 cages, although Reference #26 does provide a few examples of endohedral C28 complexes. For small cage structures with a high degree of curvature, the exohedral positioning of the metal is predominantly favored. See Comput. Theor. Chem. 2017, 1119, 32-44 and Int J. Quantum Chem. 2019, e25992 as examples. In general, the exohedrally favored position of the Ti is not necessarily startling for small cages with high curvature. What is interesting is the bonding characteristics of the Ti ion… bonded to 4 N atoms rather than just a single N atom.

We agree with the reviewer that for very small carbon cages with high curvature (like C_{20} and C_{24}, as demonstrated in the references the reviewer provided), the exohedral positioning of the metal is favored. However, we would like to stress the fact that

most of the BN fullerenes considered in this work have a relatively larger cage size, being of from 28 to 48 atoms. Our findings suggest that, for BN cages of such large sizes the exohedral positioning of the metal is still favored, whereas for carbon cages of the same sizes the opposite holds true: the endohedral positioning is preferred (as in the cases of C_{28}—C_{50}, exemplified in Reference #21). In order to make the statement more accurate, in the revised version of the manuscript (page 5) we modified the sentence as follows: "… surprising result since it is well-known that carbon metallofullerenes are generally endohedral complexes with enclosed metal atoms or clusters (at least for cage sizes as small as C_{28}), …".
